# Polygenic scores for psychiatric traits mediate the impact of multigenerational history for depression on offspring psychopathology

**DOI:** 10.1038/s41380-025-03221-8

**Published:** 2025-09-08

**Authors:** Eunji Lee, Milenna T. van Dijk, Bo-Gyeom Kim, Gakyung Kim, Eleanor Murphy, Ardesheer Talati, Yoonjung Yoonie Joo, Myrna M. Weissman, Jiook Cha

**Affiliations:** 1 Department of Psychology, Seoul National University, Seoul, South Korea; 2 Department of Psychiatry, Vagelos College of Physicians and Surgeons, Columbia University, New York, NY USA; 3 Division of Translational Epidemiology, New York State Psychiatric Institute, New York, NY USA; 4 Department of Brain and Cognitive Sciences, Seoul National University, Seoul, South Korea; 5 Department of Digital Health, Samsung Advanced Institute for Health Sciences & Technology (SAIHST), Samsung Medical Center, Sungkyunkwan University, Seoul, South Korea; 6 Department of Epidemiology, Mailman School of Public Health, Columbia University, New York, NY USA; 7 Graduate School of Artificial Intelligence, Seoul National University, Seoul, South Korea

**Keywords:** Predictive markers, Depression, Genetics, Psychology

## Abstract

A family history of depression is a well-documented risk factor for offspring psychopathology. However, the genetic mechanisms underlying the intergenerational transmission of depression remain unclear. We used genetic, family history, and diagnostic data from 11,875 9–10 year-old children from the Adolescent Brain Cognitive Development study. We estimated and investigated the children’s polygenic scores (PGSs) for 30 distinct traits and their association with a family history of depression (including grandparents and parents) and the children’s overall psychopathology through logistic regression analyses. We assessed the role of polygenic risk for psychiatric disorders in mediating the transmission of depression from one generation to the next. Among 11,875 multi-ancestry children, 8111 participants had matching phenotypic and genotypic data (3832 female [47.2%]; mean (SD) age, 9.5 (0.5) years), including 6151 [71.4%] of European-ancestry). Greater PGSs for depression (estimate = 0.129, 95% CI = 0.070–0.187) and bipolar disorder (estimate = 0.109, 95% CI = 0.051–0.168) were significantly associated with higher family history of depression (Bonferroni-corrected *P* < 0.05). Depression PGS was the only PGS that significantly associated with both family risk and offspring’s psychopathology, and robustly mediated the impact of family history of depression on several youth psychopathologies including anxiety disorders, suicidal ideation, and any psychiatric disorder (proportions mediated 1.39–5.87% of the total effect on psychopathology; FDR-corrected *P* < 0.05). These findings suggest that increased polygenic risk for depression partially mediates the associations between family risk for depression and offspring psychopathology, showing a genetic basis for intergenerational transmission of depression. Future approaches that combine assessments of family risk with polygenic profiles may offer a more accurate method for identifying children at elevated risk.

## Introduction

Depression runs in families, often manifesting in various forms of mental disorders. Parental depression increases the offspring’s risk of developing depression and other psychopathology, such as anxiety, disruptive disorders and substance use, by 2–5 times [1–3]. Children with a family history of depression often develop the disorder at a younger age, even in childhood [3]. Although the familial transmission of depression is established, the intricate mechanisms through which genetic predispositions and environmental factors contribute to the intergenerational transmission of depression and related psychopathologies are not yet fully understood [4, 5].

Offspring with both a parent and at least one grandparent with depression are at an even higher risk for developing psychopathology [6, 7]. We first found this using a longitudinal three-generational study, which used carefully crafted interview-based diagnoses for every family member, from children to adults [7–9] (Warner, Weissman et al. [9]), and these findings were confirmed by other moderate sized studies [10, 11]. We recently generalized these findings to a large, diverse cohort of preadolescents in the Adolescent Brain and Cognitive Development (ABCD) study. This study showed a significant association between family history of depression and offspring’s risk of psychopathology regardless of sociodemographic characteristics such as sex, socioeconomic status (SES), and race/ethnicity [6].

Psychiatric disorders have a high degree of heritability, estimated for depression around 30–50% [12] and up to 80% for schizophrenia [13, 14]. These numbers came initially from twin studies [15], which cannot readily distinguish between genetic and intrauterine and perinatal factors or differences between parenting monozygotic versus dizygotic twins. A recent large-scale registry study, however, confirmed the initial heritability estimates [16]. These studies suggest a strong role for genetics in the transmission of depression between generations. Nonetheless, the variance explained by genetic components is not attributed to a few candidate genes or variants, but rather to the cumulative impact of numerous variants, each with small effects. As an example, a meta-analysis involving three independent depression genome-wide association studies (GWASs), with a total sample size of 807,553 participants, identified 102 genome-wide significant variants and 269 putative genes associated with depression [17]. Polygenic scores (PGSs) represent an estimate of relative cumulative genetic risk of an individual for the phenotype of interest, such as depression, based on the findings of GWAS. PGSs for psychiatric disorders are associated with increased risk for psychopathology in the general population [18]. Some studies have examined associations between PGS and family history, but the findings have been mixed. PGS for depression was associated with a continuous score of first-degree (parents and siblings) family history loading for depression [19], but two other studies found no association between first-degree family history and depression PGS [20, 21], perhaps because they used the data from earlier and smaller GWASs. Several papers have assessed if binary forms of family history and PGSs independently predict risk for psychopathology, including depression, or complement each other, and found that family history and PGS had largely independent contributions to psychopathology, suggesting PGS may be capturing differing, or only a small fraction of, genetic risks compared to family history [22]. Risk prediction may be improved by incorporating both PGS and family history into models [23, 24]. However, as PGS capture more genetic variance, they might also explain more variance between family history and psychopathology outcomes.

Family history is not a monolith and can be assessed in a multitude of ways. Often it is used as a binary variable measuring having a parent, or any family member, with depression. We previously found that risk for psychopathology scaled with having just a grandparent, just a parent or both a parent and a grandparent with depression, with the latter leading to the largest rates of psychopathology. We therefore hypothesize that these individuals have a stronger genetic component than children with just a parent or just a grandparent with depression. To our best knowledge, no studies have yet reported whether increased family risk of depression over two generations is linked to greater polygenic risk for mental disorders, and if PGS might explain some of the increased risk for psychopathology that these high-risk children have.

In the present study, we first test whether multigenerational family risk for depression (having both a parent and a grandparent with depression) is independently associated with greater polygenic risk for psychiatric disorders. Then we test whether PGSs are associated with the presence of psychopathology. We hypothesize that as more previous generations have been affected by depression, a greater genetic risk for psychopathology and related behavioral vulnerabilities would have been accumulated in the offspring, Since psychiatric disorders are genetically correlated with non-psychiatric phenotypes such as educational attainment, subjective wellbeing, and risky behaviors [25–27], polygenic scores for a broad array of phenotypes were used to test our research questions. Lastly, we explore whether PGSs partially mediate the association between multigenerational family risk for depression and higher rates of psychiatric diagnoses in the offspring. We test this using a mediation model that incorporates depression PGS, alongside family risk and clinical data, in the ABCD study, which has the advantage of large sample size and generalizability, making it well-suited for rigorous genetic analyses.

## Methods

### Participants and data source

**Fig. 1:**
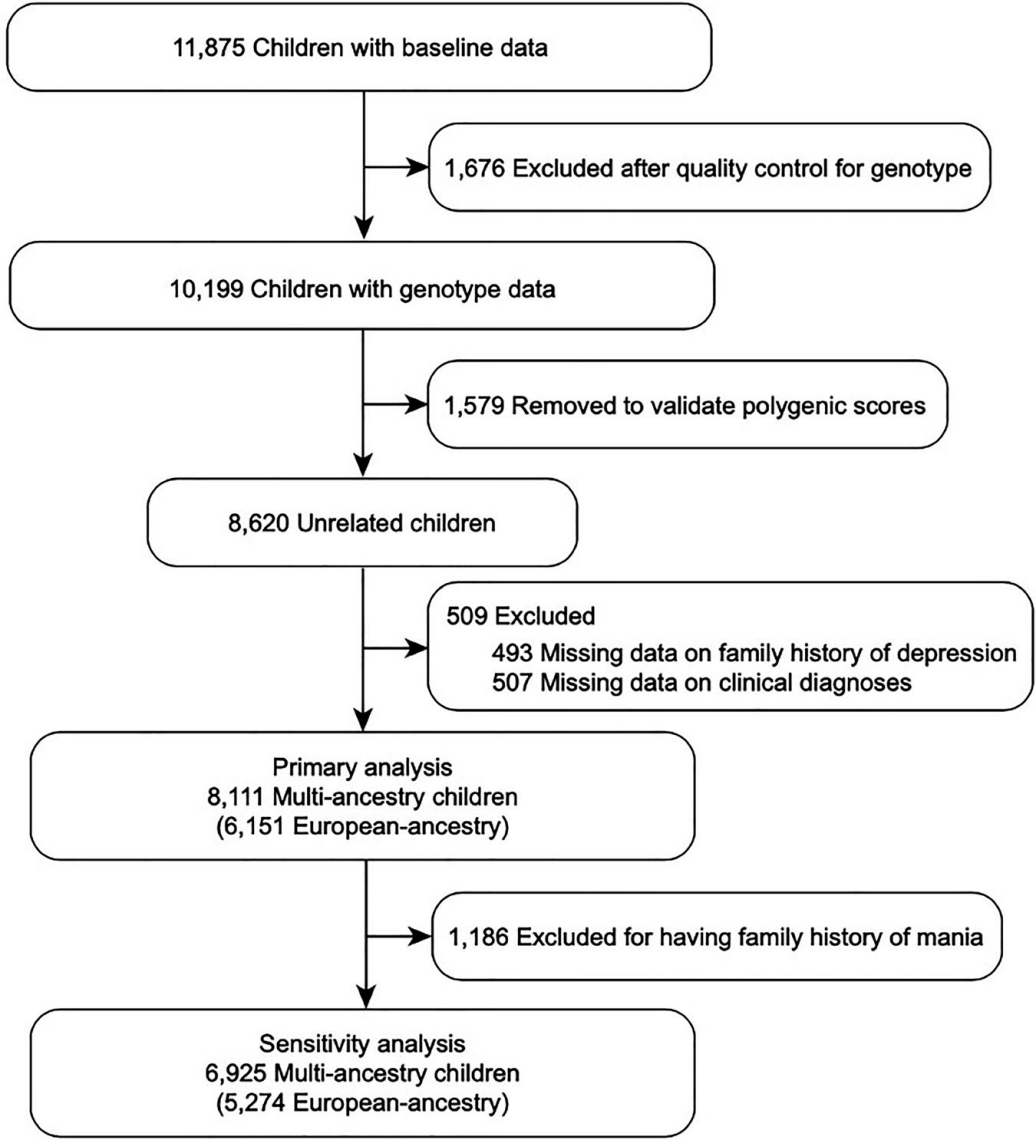
CONSORT diagram. The data from 11,875 children between the ages of 9 and 10 years old were initially evaluated, collected during the baseline visit of the Adolescent Brain and Cognitive Development study. After the quality control procedure for genotype data and removing missing values in the family history and clinical measures, the primary analysis involved examining complete phenotype, genotype, and confounder data for a multi-ancestry cohort of 8111 children, 6151 of whom were of European-ancestry. Following the exclusion of children with a family history of mania, a sensitivity analysis was conducted on a sample of 6925 multi-ancestry children, of which 5274 were of European-ancestry.

This study used baseline data of 11,875 participants aged 9–10 years from the ABCD study release 2.01, collected September 2016-November 2018 [28] across 21 research sites in the United States. The genetic ancestry of children to categorize the sample was obtained from release 3.0 (https://nda.nih.gov/study.html?id=901). All procedures for data collection were approved by the centralized institutional review board (IRB) at University of California, San Diego. Caretakers provided written informed consent and children provided assent. We imputed missing values of covariates and excluded participants without genotypes, family history, and clinical outcomes. After preprocessing genotype data and validating PGSs with a validation set, 8620 samples remained. From 8620 genotyped samples, we excluded anyone with missing values of family history (n = 493) and KSADS-5 (n = 507). The final sample (Fig. 1) included 8111 unrelated multiethnic children, consisting of 6151 [71.4%] participants of European-ancestry, 1285 [14.9%] African ancestry, 315 [3.7%] admixed American ancestry, 106 [1.2%] East Asian ancestry, and 254 [2.9%] unidentified ancestry.

### Polygenic scores

Genotyping was done using saliva samples of ABCD study participants at the baseline visit. See Supplementary Methods for Quality Assurance of the genotype data. We constructed the PGSs of 30 complex traits selected for their relationship to psychiatric disorders, using publicly available GWAS summary statistics: Attention-deficit/hyperactivity disorder (ADHD) [29], cognitive performance (CP) [30], educational attainment (EA) [30], major depressive disorder (MDD) [31], insomnia [32], snoring [32], intelligence quotient (IQ) [33], post-traumatic stress disorder (PTSD) [34], depression [17, 35], body mass index (BMI) [36, 37], alcohol dependence [38], autism spectrum disorder (ASD) [39], automobile speeding propensity (ASP) [25], bipolar disorder [40], cannabis during lifetime (cannabis use) [41], ever smoker [25], shared effects on five major psychiatric disorder (cross disorder) [42], alcoholic drinks consumption per week (drinking) [25], eating disorder [43], neuroticism [44], obsessive-compulsive disorder (OCD) [45], first principal components of four risky behaviors (risky behaviors) [25], general risk tolerance [25], schizophrenia [46, 47], worrying [44], anxiety [48], subjective well-being (SWB) [26], general happiness, and general happiness for health (happiness-health) and meaningful life (happiness-life) (http://www.nealelab.is/ukbiobank/). All GWASs were European-only samples. For depression, BMI, alcohol dependence, PTSD, and schizophrenia, non-European GWAS was also available, so a polygenic score calculated from multiple GWASs was constructed for these five traits. Therefore, for these traits, we estimated two different versions of each PGS; European GWAS-based PGSs and multiethnic GWAS-based PGSs.

The posterior effect sizes of single nucleotide polymorphisms (SNPs) were estimated using PRS-CSx [49], a Bayesian approach that enables the merging of multiple GWAS summary statistics from diverse populations. The final scores were calculated using PLINK version 1.9 and controlled for the first ten genetic principal components. The optimal hyperparameter (global shrinkage hyperparameter in PRS-CSx) for PGSs was selected in a held-out validation set of 1579 unrelated participants. These participants were genetically related to the final samples and therefore were excluded from the primary analyses. Details about the GWASs and validation procedures of PGSs are presented in Supplementary Table 1 and Supplementary Methods in Supplementary Material.

### Measures

All assessments were reported previously in van Dijk et al. published in JAMA Psychiatry [6]. The ABCD Family History Assessment was used to collect caregivers’ reports on the history of grandparents (generation 1 [G1]) and parents (generation 2 [G2]). We created a four-level depression risk variable, which describes risk levels of depression history from the two generations: (1) neither G1 nor G2 (G1−/G2−; a reference level), (2) only G1 (G1+/G2−), (3) only G2 (G1−/G2+), and (4) both G1 and G2 (G1+/G2+). Having one parent or one grandparent was sufficient to be categorized as having a parent (G2+) or grandparent (G1+) with depression. Additionally, we developed a binary “any family history” indicator to simplify the analysis. This indicator contrasts families with no reported depression history in either generation (G1−/G2−) against those with a reported history in at least one generation, so collapsing G1+/G2−, G1−/G2+, and G1+/G2+ groups into one variable “FamHist+”. Furthermore, we introduced a parental history indicator (G2− vs. G2+), specifically to highlight the impact of having a parent with a history of depression. This indicator focuses on the more immediate familial influence, distinguishing between participants without a parental history of depression (G2−) and those with such a history (G2+).

Childhood psychopathology was assessed by the Kiddie Schedule for Affective Disorders and Schizophrenia (KSADS) [50, 51] reported by parents and children. Parent and child reports were separately analyzed; therefore, a total of 36 clinical variables were included in the analyses. Further details for the measures are in the Supplementary Methods.

### Potential confounders

We used the following 13 covariates to adjust for potential confounding effects: child’s age, sex, race/ethnicity, sexual orientation reported by child and parent, gender identity, religious preference, country of birth, reporter’s relationship to child, total household income, and caregiver’s age, education level, and marital status. Multiple imputation method using the R package *‘mice’* v3.14.0 was employed to impute missing values in covariates (See Supplementary Methods).

### Statistical analysis

We primarily analyzed (using R version 4.2.0.) the data of 8111 multi-ancestry children. We repeated the analyses in 6151 European-ancestry samples to test for ancestral bias. Bonferroni and false discovery rate (FDR) corrections for the number of tests were applied to each analysis with significance set at adjusted *P* < 0.05. We centered and scaled the PGSs and continuous variables of covariates to obtain standardized estimates from regression analyses.

### Association and mediation analysis

Association between family history of depression and PGSs was assessed using univariate linear models, with the family history indicator as the independent variable and PGS as the dependent variable. Firth logistic regression was used when KSADS was the outcome with family risk and/or PGS as predictors including covariates [52]. Firth’s approach helps reduce bias in highly imbalanced data by penalizing the likelihood function. We computed 95% confidence intervals (CIs) and McFadden’s pseudo *R*^*2*^ with penalized log-likelihood [53].

For mediation analysis (using package ‘*mediation*’ v4.5.0), we selected the PGSs that were significantly associated with both clinical outcome and family history of depression and tested these as candidate mediator. The treatment variable was the familial risk of depression, considering the lowest risk (G1−/G2−) as the control condition and the others (either G1+ or G2+) as the treatment condition. Clinical outcomes that were significantly associated with both PGS and family history of depression were examined as outcome. We additionally tested the models with parental risk only. Significance of the direct effects of familial risk and the mediation effects of PGSs were estimated using bootstrap samples (n = 1000).

### Additional assessment/sensitivity analysis

Our main analyses defined depression family history only by the depression question in the Family History Assessment. However, this does not exclude individuals who might have both depression and mania (e.g. likely bipolar disorder). Therefore, we performed a sensitivity analysis (n = 6925 multi-ancestry participants, including n = 5274 European-ancestry) removing children who have parents or grandparents with mania obtained from the Family History Assessment (Fig. 1).

## Results

### Participants

The complete phenotype and genotype data from 8111 unrelated multi-ancestry children were available for analysis, including 6151 European-ancestry children. 8111 multi-ancestry children consist of 3832 [47.2%] females with a mean [SD] age at baseline of 9.48 [0.51] years. Of European-ancestry children, 2860 [46.5%] were female, and the mean [SD] age was 9.48 [0.51] years (Table 1).

**Table 1:** Demographics and family history of depression for multi-ancestry and European-ancestry participants.

	Multi-ancestry (n = 8111)		European-ancestry (n = 6151)		Test statistics	
Child’s demographics	N (%)	Mean (SD)	N (%)	Mean (SD)	*t / χ*^*2*^	*P*
Age		9.48 (0.51)		9.48 (0.51)	0.119	0.905
Sex						
Male	4279 (52.76)		3291 (53.50)		0.786	0.375
Female	3832 (47.24)		2860 (46.50)			
Race/Ethnicity						
Hispanic	1772 (21.85)		1204 (19.57)		958.460	<0.001
Non-Hispanic black	1203 (14.83)		45 (0.73)			
Non-Hispanic white	4996 (61.60)		4833 (78.57)			
Other	140 (1.73)		69 (1.12)			
Sexual Orientation (parent report)						
Yes	3 (0.04)		1 (0.02)		13.141	0.004
Maybe/Don’t know	648 (7.99)		595 (9.67)			
No	7390 (91.11)		5499 (89.40)			
Decline to answer	70 (0.86)		56 (0.91)			
Sexual Orientation (child report)						
Yes	31 (0.38)		26 (0.42)		2.338	0.505
Maybe	72 (0.89)		61 (0.99)			
No	6006 (74.05)		4488 (72.96)			
I do not understand this question	2002 (24.68)		1576 (25.62)			
Gender identity (parent report)						
Male	4276 (52.72)		3288 (53.45)		1.062	0.900
Female	3828 (47.20)		2856 (46.43)			
Trans male	0 (0.00)		0 (0.00)			
Trans female	2 (0.02)		2 (0.03)			
Gender queer	1 (0.01)		1 (0.02)			
Other identity	4 (0.05)		4 (0.07)			
Religious preference						
Agnostic/Atheist	379 (4.67)		366 (5.95)		15.863	<0.001
Denominational	5889 (72.61)		4316 (70.17)			
Non-denominational	1843 (22.72)		1469 (23.88)			
Country of birth						
USA and territories	7887 (97.24)		5994 (97.45)		0.589	0.443
Other	224 (2.76)		157 (2.55)			
**Caregiver’s demographics**	**N (%)**	**Mean (SD)**	**N (%)**	**Mean (SD)**	***t / χ***^***2***^	***P***
Relationship with child						
Biological mother	6970 (85.93)		5284 (85.90)		12.389	0.0147
Biological father	803 (9.90)		668 (10.86)			
Adoptive parent	158 (1.95)		85 (1.38)			
Custodial parent	78 (0.96)		45 (0.73)			
Other	102 (1.26)		69 (1.12)			
Age		39.96 (6.78)		40.72 (6.36)	0.123	0.902
Marital status						
Married	5537 (68.27)		4727 (76.85)		211.720	<0.001
Widowed	60 (0.74)		41 (0.67)			
Divorced	742 (9.15)		571 (9.28)			
Separated	305 (3.76)		189 (3.07)			
Never married	968 (11.93)		342 (5.56)			
Living with partner	499 (6.15)		281 (4.57)			
Household income		7.16 (2.44)		7.77 (2.01)	0.736	0.462
Parental education		16.63 (2.75)		17.18 (2.34)	1.037	0.300
Family history of depression						
G1−/G2−	4658 (57.43)		3284 (53.39)		30.870	<0.001
G1+/G2−	1092 (13.46)		967 (15.72)			
G1−/G2+	882 (10.87)		650 (10.57)			
G1+/G2+	1479 (18.23)		1250 (20.32)			

G1, generation 1; G2, generation 2.

### Association between familial risks and polygenic scores

First, we tested whether multigenerational family risk for depression is associated with greater polygenic risk. Our primary analysis included all children (a multi-ancestry sample), and our supporting analysis included only European-ancestry children to test for potential bias related to ancestry. Our analyses using linear regression without adjusting for potential confounders demonstrated a significant association between multigenerational family risk for depression and increased PGSs for depression (estimate, 0.143 [95% CI, 0.084–0.201]), neuroticism (estimate, 0.112 [95% CI, 0.053–0.170]), and bipolar disorder (estimate, 0.108 [95% CI, 0.050–0.167]; Bonferroni-corrected *P* < 0.05; Supplementary Fig. 1A). Specifically, children from the highest familial risk category (G1+/G2+) displayed significantly elevated polygenic risks compared to those from the lowest risk category (G1−/G2−). However, no significant differences in PGS were observed among children with a history of depression in either parents or grandparents (FamHist+), as opposed to those with the lowest familial risk (G1−/G2−). When isolating parental history regardless of grandparental history (G2− vs. G2+), children with a parental history of depression showed significantly higher polygenic risks for depression (estimate, 0.103 [95% CI, 0.055–0.150]), neuroticism (estimate, 0.094 [95% CI, 0.046–0.141]), ever been a smoker (estimate, 0.082 [95% CI, 0.034–0.130]), and BMI (estimate, 0.081 [95% CI, 0.033–0.129]) compared to those without such a history (Supplementary Fig. 1C). These findings demonstrate that the effect sizes associated with multigenerational history for PGS are larger than those observed for parental history alone. Additionally, the same analyses conducted exclusively with European-ancestry children yielded results consistent with those from the multi-ancestry children (Supplementary Fig. 2).

**Fig. 2:**
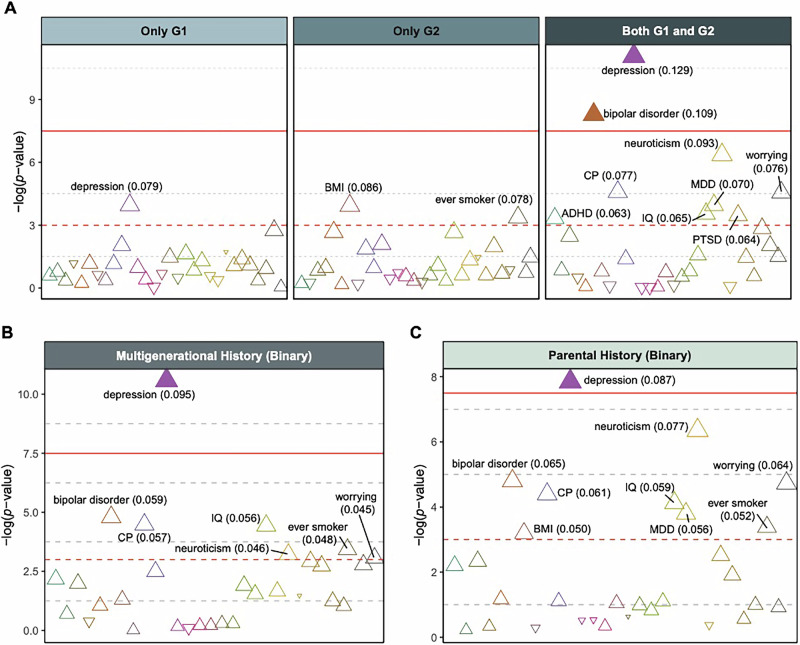
Effects of family history of depression on residualized polygenic scores in multi-ancestry children. Dashed red line indicates 0.05 of unadjusted *P*. Solid red line indicates 0.05 of Bonferroni-corrected *P*. Each triangle represents a PGS with the odds ratio (OR) of family risk. Triangles filled with color denote PGSs with FDR-corrected *P* < 0.05. *P* values were adjusted for 30 tests. **A** Regression of four-level family risk from the two generations: neither G1 nor G2 (G1−/G2−; reference level), only G1 (G1+/G2−), only G2 (G1−/G2+), and both G1 and G2 (G1+/G2+). Detailed information underlying this figure are available in Supplementary Table 2. **B** Regression of binary any family history: neither G1 nor G2 (G1−/G2−; reference level) and the rest of the groups (FamHist+). Detailed information underlying this figure are available in Supplementary Table 5. **C** Regression of parental depression history: no history in the parent (G2–; reference level) and depression in the parent (G2+). Detailed information underlying this figure are available in Supplementary Table 6. ADHD attention-deficit/hyperactivity disorder, MDD major depressive disorder, BMI body mass index, EA educational attainment, CP cognitive performance, IQ intelligence quotient, PTSD post-traumatic stress disorder.

Follow-up linear regression analyses revealed that family risk remained significantly associated with the PGSs for depression and bipolar disorder, after accounting for the potential confounding variables. Specifically, children with the highest familial risk exhibited significantly higher polygenic risk compared to those with the lowest risk (G1−/G2− vs. G1+/G2+; estimate for depression PGS, 0.129 [95% CI, 0.070–0.187]; estimate for bipolar disorder PGS, 0.109 [95% CI, 0.051–0.168]; Bonferroni-corrected *P* < 0.05; Fig. 2A; Supplementary Table 2). To further examine potential differences between multigenerational depression risk categories, we conducted additional Z-tests comparing the coefficients of G1+/G2−, G1−/G2+, and G1+/G2+ on PGSs for depression and bipolar disorder. The results showed no statistically significant differences in most comparisons. For depression PGS in multi-ancestry children (Supplementary Table 2), the comparisons between G1+/G2− and G1−/G2+ (Z = 0.446, *P* = 0.656) and between G1+/G2− and G1+/G2+ (Z = −1.117, *P* = 0.264) were not statistically significant. A similar pattern was observed for bipolar disorder PGS, though the comparison between G1−/G2+ and G1+/G2+ yielded a marginally significant result (Z = −2.159, *P* = 0.031).

To determine if genetic risk increased as children had more familial risk, similar to a dose-response relationship, we tested whether family risk of depression, treated as a continuous predictor, was significantly associated with PGSs. In the multi-ancestry children (Supplementary Table 3), the continuous family risk variable had a significant effect on depression PGS (estimate, 0.041 [95% CI, 0.022–0.059], *P* = 1.34E-05, Bonferroni-corrected *P* < 0.05). Significant associations were also observed for bipolar disorder, neuroticism, CP, worrying, IQ, major depressive disorder, PTSD, and ever smoker PGSs (uncorrected *P* < 0.05). Similar results were found when restricting the analysis to European-ancestry children (Supplementary Table 4), with the continuous risk variable significantly predicting depression PGS (estimate, 0.041 [95% CI, 0.021–0.062], *P* = 8.81E-05, Bonferroni-corrected *P* < 0.05) and showing significant associations with bipolar disorder, CP, IQ, neuroticism, and worrying PGSs (uncorrected *P* < 0.05).

The binary any multigenerational family history (G1−/G2− vs. FamHist+) had a significant effect on depression PGS (estimate, 0.095 [95% CI, 0.051–0.139]; Bonferroni-corrected *P* < 0.05; Fig. 2B; Supplementary Table 5). Any family history was not significantly associated with the other PGSs. Likewise, when considering only parental depression history but not grandparents’ history, the depression PGS was significantly associated with having a parent with depression (estimate, 0.087 [95% CI, 0.039–0.135]; Bonferroni-corrected *P* < 0.05; Fig. 2C; Supplementary Table 6). Models with European-ancestry children alone showed similar results (G1−/G2− vs. G1+/G2+; estimate for depression PGS, 0.136 [95% CI, 0.071–0.201]; estimate for bipolar disorder PGS, 0.126 [95% CI, 0.061–0.191]; Bonferroni-corrected *P* < 0.05; Supplementary Fig. 3).

As a sensitivity analysis, we excluded children with family history of mania and repeated the analyses. With the four-level family risk, depression PGS showed a significant association, but bipolar disorder PGS did not (estimate, 0.115 [95% CI, 0.047–0.182]; FDR-corrected *P* < 0.05, not Bonferroni-significant; Supplementary Fig. 4A). Likewise, binary any family history showed a significant association with depression PGS (estimate, 0.095 [95% CI, 0.047–0.143]; Bonferroni-corrected *P* < 0.05; Supplementary Fig. 4B). No significant association was found in the regression models with parental depression history (Supplementary Fig. 4C). However, when analyses were confined to children of European-ancestry, excluding those with a history of mania, no significant associations were observed (Supplementary Fig. 5).

### Association between psychopathology and polygenic scores

**Fig. 3:**
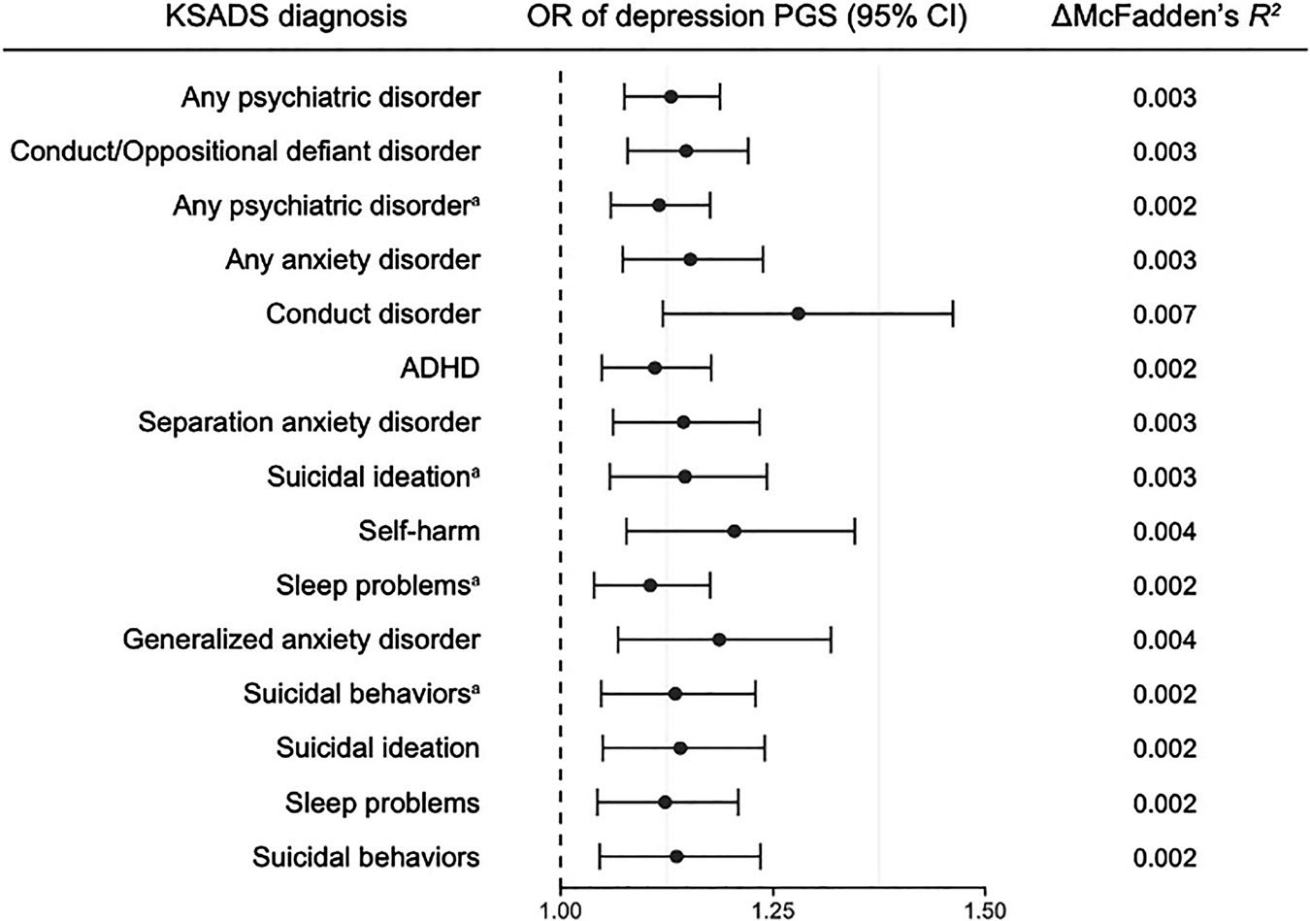
Effects of depression PGS on clinical outcomes in multi-ancestry children. ^a^reported by child; otherwise reported by parent. Presented results in the figure were from the models with significant effects of depression PGS after the FDR correction. No significant result was found with bipolar disorder PGS. Error bar indicates 95% confidence interval. *P* values were adjusted for 72 tests (36 outcomes and 2 PGSs of depression and bipolar disorder). Detailed information underlying this figure are available in Supplementary Table 7. ΔMcFadden’s *R*^*2*^, the proportion of variance explained by polygenic score; ADHD, attention-deficit/hyperactivity disorder.

We investigated whether the PGSs associated with family history of depression would similarly be associated with offspring’s psychopathology. Depression PGS was positively associated with parent (OR, 1.130 [95% CI, 1.075–1.188]) and child reports (OR, 1.116 [95% CI, 1.059–1.176]) of any psychiatric disorder, conduct/oppositional defiant disorder (OR, 1.148 [95% CI, 1.079–1.221]), parent report of any anxiety disorder (OR, 1.153 [95% CI, 1.073–1.238]), conduct disorder (OR, 1.280 [95% CI, 1.120–1.462]), ADHD (OR, 1.111 [95% CI, 1.049–1.177]), separation anxiety disorder (OR, 1.145 [95% CI, 1.062–1.234]), parent (OR, 1.141 [95% CI, 1.050–1.240]) and child reports (OR, 1.146 [95% CI 1.058–1.243]) of suicidal ideation (FDR-corrected *P* < 0.05; Fig. 3; Supplementary Table 7). However, bipolar disorder PGS was not associated with any clinical outcomes (unadjusted *P* > 0.05). Within the regression models, the variances explained by depression PGS ranged from 0.15 to 0.65%. In European-ancestry children, depression PGS was also positively associated with behavioral problems, suicidal behaviors, and any psychiatric disorder (FDR-corrected *P* < 0.05; Supplementary Fig. 6; Supplementary Table 8). Additionally, when testing associations between family history of depression and KSADS diagnoses, we observed that the risk for psychiatric disorders and suicidal behaviors increases as family risk increases both in multi-ancestry and European-ancestry children (Supplementary Tables 9–10), similar to what we have found previously [6].

With the four-level family risk variable included in the models with depression PGS, PGS for depression was still significantly associated with KSADS diagnoses such as any psychiatric disorder, conduct/oppositional defiant disorder, conduct disorder, any anxiety disorder, sleep problems, and suicidal ideation (FDR-corrected *P* < 0.05; Supplementary Table 11). The models including both depression PGS and family risk accounted for a slightly greater variance of KSADS diagnoses (parent-reported any psychiatric disorder; 2.59%) than the model including only family history of depression (2.41%). Likewise, in European-ancestry samples, depression PGS showed significant effects on similar KSADS diagnoses including both family history and depression PGS (FDR-corrected *P* < 0.05; Supplementary Table 12).

### Mediation analysis with PGSs as mediators

Given that only the depression PGS was found to be significantly associated with both familial depression history and offspring’s psychopathology, we further investigated whether depression PGS mediated the effects of familial depression history on offspring’s psychopathology.

**Fig. 4:**
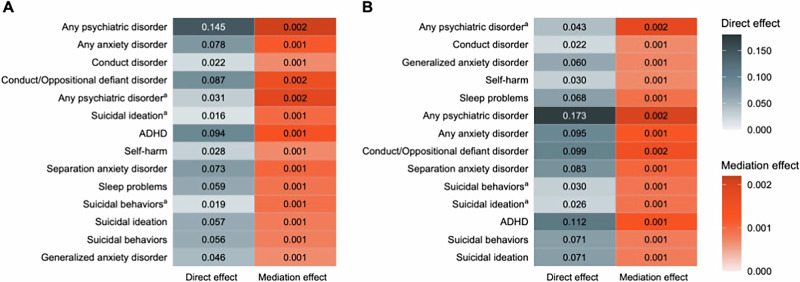
Mediation analysis with depression PGS in multi-ancestry children. ^a^reported by child; otherwise reported by parent. **A** Multigenerational family history of depression as treatment variable (G1−/G2− vs. FamHist+). **B** Parental history of depression as treatment variable (G2− vs. G2+). All tested effects were with FDR-corrected *P* < 0.05. *P* values were adjusted for 28 tests (2 versions of family history and 14 clinical outcomes).

The mediation models with the binary indicator of any family history (G1−/G2− vs. FamHist+) showed that depression PGS had significant mediation effects on all tested 14 clinical outcomes including parent- (estimate, 0.0021 [95% CI, 0.0009–0.0037]) and child-reported (estimate, 0.0019 [95% CI, 0.0008–0.0033]) any psychiatric disorders, any anxiety disorders (estimate, 0.0012 [95% CI, 0.0005–0.0007]), conduct/oppositional defiant disorder (estimate, 0.0016 [95% CI, 0.0006–0.0027]), ADHD (estimate, 0.0014 [95% CI, 0.0005–0.0018]), self-harm (estimate, 0.0007 [95% CI, 0.0002–0.0013]), sleep problems of parent report (estimate, 0.0009 [95% CI, 0.0002–0.0018]), and suicidal behaviors of child (estimate, 0.0009 [95% CI, 0.0002–0.0018]) and parent reports (estimate, 0.0008 [95% CI, 0.0002–0.0016]; FDR-corrected *P* < 0.05; Fig. 4A). The proportions mediated by depression PGS relative to the total effects on each clinical outcome ranged from 1.39% to 5.87%. Similar results were observed with the mediation models of parental depression history. Depression PGS had significant mediation effects on all 14 KSADS diagnoses, except for parent report of suicidal ideation (Fig. 4B). Likewise, in European-ancestry samples, depression PGS significantly mediated the impact of multigenerational and parental family risk on KSADS diagnoses (Supplementary Fig. 7).

## Discussion

Familial history of depression increases risk for offspring psychiatric disorder. Therefore, we investigated whether polygenic risks for psychiatric disorders contribute to intergenerational transmission of depression. We found that multigenerational depression history was positively correlated with higher genome-wide PGSs for depression and bipolar disorder. Moreover, a greater PGS for depression significantly mediated the effects of familial history of depression on offspring’s mental health indexed by KSADS diagnoses. To our best knowledge, this is the first report showing polygenic risk for depression as a partial mediator for the transgenerational transmission of depression.

Addressing conflicting results from previous research on the association between first-degree family history and depression PGS [19–21], the present study utilized more recent and larger GWAS results for constructing the PGS of depression, as well as using a multigenerational family history measure, which could partially account for the discrepancies between the findings as well as testing findings in a larger cohort. For the first time, our results reveal that higher depression PGS is significantly associated with a family history of depression spanning multiple generations, encompassing risks from both first- and second-degree family members. The children with two previous generations affected showed the highest PGS (estimate, 0.129 [95% CI, 0.070–0.187]) in line with the increased risk for psychopathology [6]. These offspring with two previous generations affected might form a subgroup of individuals with an exceptionally high genetic burden.

We found a similar association for bipolar PGS (estimate, 0.109 [95% CI, 0.051–0.168]). But when we limited the sample to those with a family history of depression *without* mania, bipolar PGS lost its significance in the association with family history. This shows that the significant associations with bipolar PGS might be due to including children with a family history of bipolar disorder and that genetic risk for and familial aggregation of depression and bipolar disorder are separate. This is in line with recent literature showing an association between family history of bipolar disorder and higher bipolar PGS in offspring [54–56], reports of familial transmission being specific to type of mood disorder [57] and genetic differences between depression and bipolar disorder [58].

Our results also showed that depression PGS and family history of depression together accounted for significant variances of offspring’s psychopathology. Previous findings from recent studies that showed family history and PGS are predictive of future cases of depression with shared and independent contributions, and including both factors in a model modestly increases the prognostic power in predicting depression [59–61]. The present results extend these findings by incorporating multifamily history in a large cohort of children and showing associations between multigenerational history, PGSs, and increased psychopathology through partial mediation. Previous studies investigating family history, PGS and psychopathology did not directly test associations between PGS and family history, nor did they incorporate multigenerational family history. Our objective was not primarily to investigate the independent and joint contributions of PGS and family history to psychopathology outcomes but rather whether increased genetic risk as measured by PGS could partially account for the increased rates of psychopathology we previously found in children who had both a parent and grandparent with depression.

The depression PGS was positively correlated with a wide range of KSADS diagnoses, including any psychiatric disorder, any anxiety disorder, suicidal ideation, ADHD, and conduct/oppositional defiant disorder (C/ODD). On the other hand, bipolar PGS showed no significant correlations with KSADS diagnoses. Despite not surviving correction for multiple comparisons, depression PGS was associated with any depressive disorder even at this young age of 9 to 10 years old (unadjusted *P* = 0.019). Depression often starts later in adolescence and early adulthood, but higher family and polygenic risk of depression have been shown to be associated with earlier onset [3, 62]. Suicidal ideation and self-harm at this age, also associated with depression PGS, are becoming more common in recent years [63] and are predictive of later suicide attempts [64]. However, identifying those at risk for suicide is challenging. Our results indicate that the combination of family assessments and PGS for depression might increase predictive power in evaluating risk for suicidal thoughts and behaviors in children, especially as PGS are thought to be able to explain more variance in the future as GWASs include ever larger samples.

PGS for depression significantly mediates the effects of family history on offspring’s depression. Moreover, the mediation effect was significant for a broad range of psychiatric disorders, including any psychiatric disorder, any anxiety disorder, suicidal thoughts and behaviors, C/ODD, and ADHD. This highlights a role for genetic liability in intergenerational transmission of childhood psychopathology in general and depression specifically in periadolescent children (9–10 years old). Future research may examine the brain correlates of family history of depression and depression PGS.

Family history of depression may affect the offspring’s risk for psychiatric disorders through genetic or environmental pathways. Here we show that the genetic component is significant and scales with the number of generations affected. On the other hand, children of parents with depression may experience and even generate more stressful life events [65, 66]. These events may exacerbate risks of psychiatric disorders. Further research could identify protective or vulnerability factors that may moderate the impact of family history of depression and depression PGS through gene-environment interactions. Furthermore, because we did not have access to parent and grandparent genotypes, we are unable to distinguish between direct genetic effects of the child’s genotype on their phenotype and indirect genetic effects through genetic nurture and other mechanisms. For example, the parent and the child may share a genotype associated with higher neuroticism, which could lead to less affectionate caregiving of the parent to the child [67, 68]. This, in turn, might bias our estimates of the direct effect of the neuroticism PGS on child psychiatric outcomes through altered parenting.

### Strengths and limitations

The current study has the strengths of a large, diverse multi-ancestry sample, multigenerational family history assessment, and the latest Bayesian polygenic score estimation method to test our hypotheses. Limitations of this study include that in most GWASs, the PGSs were derived from European-ancestry samples and, therefore, the generalizability of the summary statistics to other ancestry samples might be limited. Nevertheless, our findings were consistent across the analyses of multi-ancestry and European-only samples. In addition, PGSs still only account for relatively small variances compared to the heritability of depression estimated from twin studies or DNA, but PGS may become stronger predictors in future. Family history was assessed retrospectively by the caregiver, which may bias reports and underestimate effect sizes compared to samples with gold-standard clinician-based diagnoses for all family members. However, this limitation is mitigated by the fact that we previously showed that clinical results were similar between an interview-based design study [7, 8] and the ABCD study [6]. In our prior clinical studies, only the lowest- and highest-risk groups showed a statistically significant difference in diagnosis rates, but using Cochran-Armitage Test for Trend we showed statistically significant linearly increasing psychopathology with greater family risk [6]. Similarly, in this study, polygenic scores differed significantly only between the highest- and lowest-risk categories, yet the coefficients increased with higher family risk (Supplemental Table 2), and when using familial risk as a continuous predictor (as the Cochran-Armitage Test cannot be used for continuous outcomes) we also found significant associations with PGS, suggesting a dose-response relationship. This finding implies that genetic risk accumulates across generations, with each additional affected generation incrementally contributing to overall psychopathology. Lastly, the effect sizes that we found, while similar to those of prior polygenic score studies [69, 70] are small, suggesting that these PGS do not yet have high clinical significance. As GWAS discovery samples become larger and include multiple genetic ancestries, clinical significance may improve. On the other hand, the children in our sample are still young and before the median age of onset of depression. Some of the currently unaffected children might develop psychiatric disorders as the cohort ages, forming a stronger association between PGS and psychiatric disorders, and increasing the effect sizes.

In summary, we show that PGSs for depression and bipolar disorder are associated with family history of depression and that depression PGS mediates part of the association between (multigenerational) family history and offspring’s psychopathology. We demonstrate that having more previous generations affected with depression is linked to having higher polygenic risk for psychiatric disorders. The findings also implicate polygenic risk for depression as a potential mechanism for intergenerational transmission of depression, suggesting that integrating depression PGS with depression history may aid in identifying children at higher risk for psychopathology.

## Supplementary information

Supplementary Methods and Figure

## Data Availability

ABCD Dataset: https://nda.nih.gov/abcd/request-access. All GWAS summary statistics used to derive the polygenic scores are publicly available through the original publications cited in Supplementary Data 1.
