# Supplementary material for: Polygenic scores for psychiatric traits mediate the impact of multigenerational history for depression on offspring psychopathology
Source: Mol Psychiatry. 2025 Sep 8;31(2):1074–84. doi: 10.1038/s41380-025-03221-8 (PMC12815659; doi:10.1038/s41380-025-03221-8)
Supplement: Supplementary file 1 — Supplementary Methods and Figure [file 41380_2025_3221_MOESM1_ESM.pdf]

Supplementary Information

Polygenic scores for psychiatric traits mediate the impact of multigenerational history for depression on offspring psychopathology

Eunji Lee*, Milenna T. van Dijk*, Bo-gyeom Kim, Gakyung Kim, Eleanor Murphy, Ardesheer Talati, Yoonjung Y. Joo, Myrna M. Weissman, Jiook Cha

(* denotes equal contributions)

Supplementary Methods. Genotype data and measures

Supplementary Figure 1. Effects of family history of depression on original PGSs in multi-ancestry children

Supplementary Figure 2. Effects of family history of depression on original PGSs in European-ancestry children

Supplementary Figure 3. Effects of family history of depression on residualized PGSs in European-ancestry children

Supplementary Figure 4. Effects of family history of depression on residualized PGSs in multi-ancestry children (without family history of mania)

Supplementary Figure 5. Effects of family history of depression on residualized PGSs in European-ancestry children (without family history of mania)

Supplementary Figure 6. Effects of depression PGS on clinical outcomes in European-ancestry children

Supplementary Figure 7. Mediation analysis with depression PGS in European-ancestry children

Supplementary References

Supplementary Methods. Genotype data and measures

Genotype Data

Genotyping was done using saliva samples of ABCD study participants at the baseline visit. The samples were genotyped at the Rutgers University Cell and DNA Repository using the Affymetrix NIDA Smokescreen array (733,293 SNPs). We removed any SNPs with genotype call rate<95%, sample call rate<95%, and rare variants with minor allele frequency (MAF)<0.01. The variants were imputed with the Michigan Imputation Server (Das et al., 2016) using the 1000 Genome phase 3 version 5 multiethnic GRCh37/hg19 reference panel (Auton et al., 2015) with Eagle v2.4 phased output (Loh et al., 2016). With the imputed 12,046,090 variants, we additionally filtered out the data with any individuals with>5% missing genotypes; with extreme heterozygosity (F coefficient>3 SDs from the population mean); and SNPs with imputation quality INFO score<0.4, missingness rate>5%, MAF<0.01 and Hardy-Weinberg equilibrium $p<10^{-6}$. The ABCD release 3.0 provided the genetic ancestry of each participant, determined with the fastSTRUCTURE algorithm (Raj et al., 2014). Considering that the samples had diverse genetic ancestries and included related family members, we estimated kinship coefficients (KCs) and ancestrally informative principal components (PCs) using PC-Air (Conomos et al., 2015) and PC-Relate (Conomos et al., 2016) to control familial relatedness and admixed ancestry. In addition, we retained genetically unrelated participants more distant than 4th-degree relatives (KC>0.022) and removed any outliers deviating significantly from the center of PC space (>6 SD limits). We finally used total genotype data of 11,301,999 variants in 10,199 unrelated multiethnic samples, including 7,893 European-ancestry participants.

Validation of polygenic scores

For 14 PGS traits (i.e., depression, MDD, ADHD, general happiness, happiness-health, happiness-life, SWB, insomnia, snoring, BMI, PTSD, CP, EA, IQ) that have related phenotypes measured in the ABCD study, we manually validated for the optimal hyperparameter of PGSs. The global shrinkage hyperparameter (phi, φ) in the PRS-CSx was optimized with small-scale grid search (φ = 1, 1E-2, 1E-4, 1E-6) in the held-out validation set of 1,579 unrelated participants, who were excluded from the final samples. We validated the hyperparameter with linear models, regressing phenotype variables related to complex traits of each PGS. The regression models included sex, age, and the first ten ancestrally informative PCs as covariates, and the model performance was evaluated with the R^2 and beta coefficient of PGS. The rest of 16 PGS traits were validated automatically using PRS-CS-auto (Ge et al., 2019) that select the optimal value of hyperparameter with a fully Bayesian method. The final PGSs were residualized with the first ten genetic PCs for population stratification.

Potential confounders

Variable	Description	Number of missing values	Imputation method
CHILD'S CHARACTERISTICS			
demo_brthdat_v2	Age	11	predictive mean matching
demo_sex_v2	Sex	1	logistic regression
demo_ethn_v2 (Hispanic), demo_race_a_p___10 (White), demo_race_a_p___11 (Black)	Race/Ethnicity	106	polytomous logistic regression
kbi_p_c_gay	Sexual orientation (parent report)	6	polytomous logistic regression
kbi_y_sex_orient	Sexual orientation (child report)	14	polytomous logistic regression
demo_gender_id_v2	Gender identity (parent report)	12	polytomous logistic regression

demo_relig_v2	Religious preference[a]	414	polytomous logistic regression
demo_origin_v2	Country of birth[b]	12	logistic regression
PARENT/CAREGIVER'S CHARACTERISTICS			
demo_prim	Relationship with child	1	polytomous logistic regression
demo_prnt_age_v2	Age	62	predictive mean matching
demo_prnt_marital_v2	Marital status	75	polytomous logistic regression
demo_comb_income_v2	Total household income	717	predictive mean matching
demo_prnt_ed_v2	Parental education level	12	predictive mean matching

[a]Religious preference: A variable with three categories was created from the scratch variable with 17 categories ('*demo_relig_v2*') into (1) "Denominational" from categories 1-13 and 16, (2) "Non-denominational" from category 17, and (3) "Agnostic/Atheist" from categories 14-15.

[b]Country of birth: A variable with two categories was created from the scratch variable of parents' place of birth ('*demo_origin_v2*') into (1) "Born in USA and territories" from category 189, (2) "Foreign-born" from all other categories.

Family history of depression

Family history variable was created from ABCD Family HIstory Assessment-Part I ('*fhxp102*'), adapted from van Dijk et al. (2021) (Van Dijk et al., 2021). The question for depression history was "Has ANY blood relative of your child ever suffered from depression, that is, have they felt so low for a period of at least two weeks that they hardly ate or slept or couldn't work or do whatever they usually do?". If at least one of four scratch variables for paternal and maternal grandparents ('*fam_history_q6b_depression*', '*fam_history_q6c_depression*', '*fam_history_q6e_depression*', '*fam_history_q6f_depression*') has been coded 'yes', generation 1[G1] was considered as having depression. Likewise, if at least one of two scratch variables for biological father and mother ('*fam_history_q6a_depression*', '*fam_history_q6d_depression*'), generation 2 [G2] was considered as having depression.

Child psychiatric disorders

Each variable for lifetime diagnosis of psychiatric disorder was coded dichotomously from Kiddie Schedule for Affective Disorders and Schizophrenia (KSADS) of parent ('*abcd_ksads01*') and child ('*abcd_ksads501*') reports. Nine psychiatric disorders reported by both parent and child (a total of 18 variables; major depressive disorder (MDD), any depressive disorder (including MDD, persistent depressive disorder, and depressive disorder not otherwise specified), social anxiety disorder (SAD), generalized anxiety disorder (GAD), any anxiety disorder (including GAD, SAD, and specific anxiety disorder not meeting criteria for GAD or SAD), bipolar disorder, sleep problems, suicidal behaviors (including active or passive ideation, plans/intention/preparation, and attempts), and any psychiatric disorder/condition) and eight reported only by parent (agoraphobia, simple/specific phobia, separation anxiety disorder, obsessive-compulsive disorder (OCD), post-traumatic stress disorder (PTSD), attention-deficit hyperactivity disorder (ADHD), conduct/oppositional defiant disorder (meeting criteria for conduct disorder or oppositional defiant disorder), and psychotic disorder) were adapted from van Dijk et al. (2021) (Van Dijk et al., 2021).

Additionally, we included suicidal plan, suicidal ideation, and suicidal attempt reported by parents and children, and self-harm, eating disorder, conduct disorder, panic disorder reported by parents only. A total of 36 variables were used in this study.

Supplementary Figure 1. Effects of family history of depression on original PGSs in multi-ancestry children

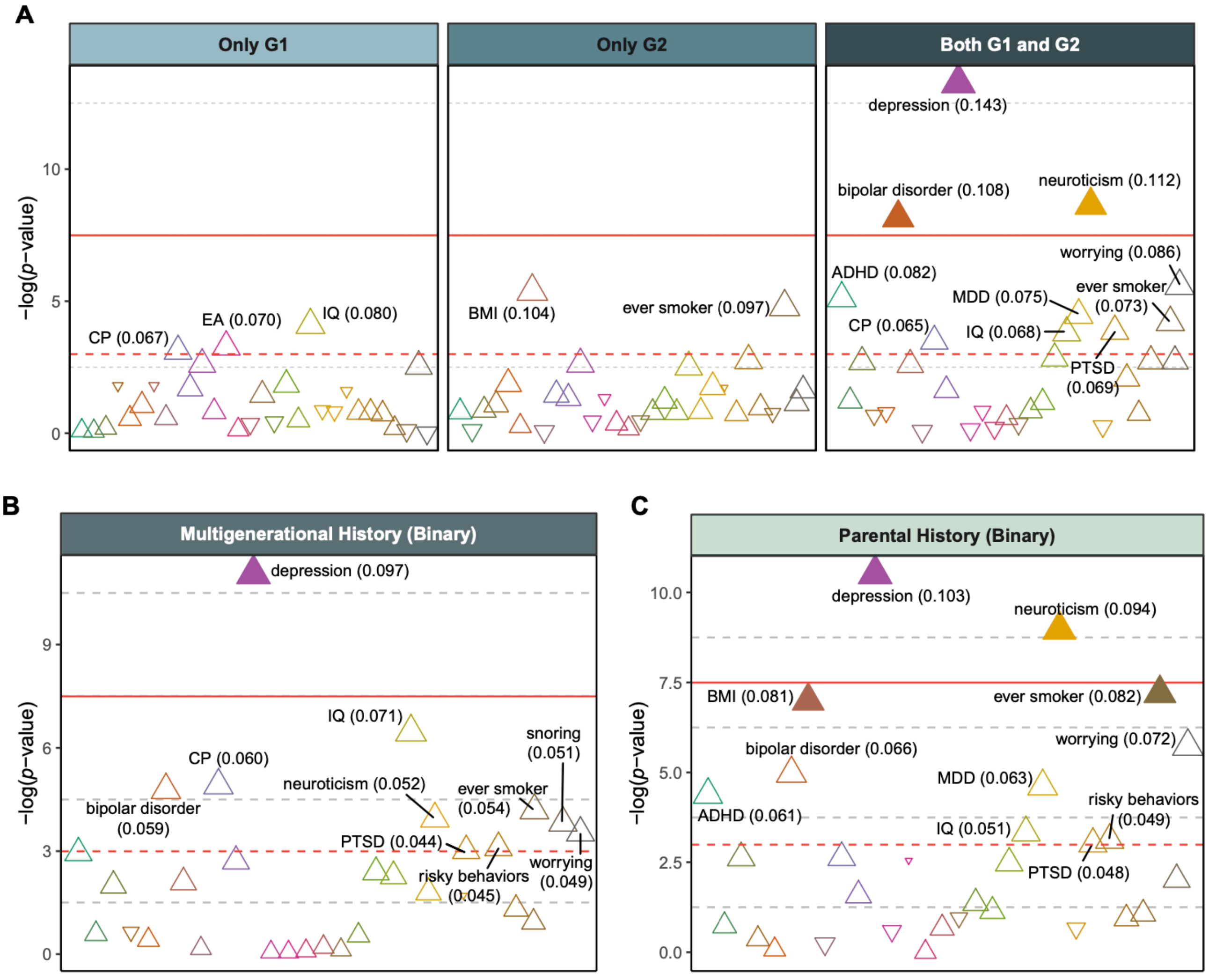

Dashed red line indicates 0.05 of unadjusted *P*. Solid red line indicates 0.05 of Bonferroni-corrected *P*. Each triangle represents a PGS with the odds ratio (OR) of family risk. Triangles filled with color denote PGSs with FDR-corrected *P*<.05. *P* values were adjusted for 30 tests. **(A)** Regression of four risk levels of family history from the two generations: no depression history (G1−/G2−; reference level), only grandparent (G1+/G2−), only parent (G1−/G2+), and both generations (G1+/G2+). Additional Z-tests were conducted to compare coefficients. For Depression PGS, the Z-test results were as follows: G1+/G2− vs. G1−/G2+, Z = −0.114, *P* = 0.909; G1+/G2− vs. G1+/G2+, Z = −1.841, *P* = 0.066; and G1−/G2+ vs. G1+/G2+, Z = −1.624, *P* = 0.104. For Neuroticism PGS, the results were: G1+/G2− vs. G1−/G2+, Z = −1.121, *P* = 0.262; G1+/G2− vs. G1+/G2+, Z = −3.892, *P* < 0.001; and G1−/G2+ vs. G1+/G2+, Z = −0.869, *P* = 0.385. For Bipolar Disorder PGS, the results were: G1+/G2− vs. G1−/G2+, Z = 0.194, *P* = 0.846; G1+/G2− vs. G1+/G2+, Z = −0.708, *P* = 0.479; and G1−/G2+ vs. G1+/G2+, Z = −3.029, *P* = 0.002. **(B)** Regression of two-level familial risk: no depression history (G1−/G2−; reference level) and the rest of the groups (FamHist+). **(C)** Regression of parental depression history: no history in the parent (G2−; reference level) and depression in the parent (G2+). ADHD, attention-deficit/hyperactivity disorder; MDD, major depressive disorder; BMI, body mass index; EA, educational attainment; CP, cognitive performance; IQ, intelligence quotient; PTSD, post-traumatic stress disorder.

Supplementary Figure 2. Effects of family history of depression on original PGSs in European-ancestry children

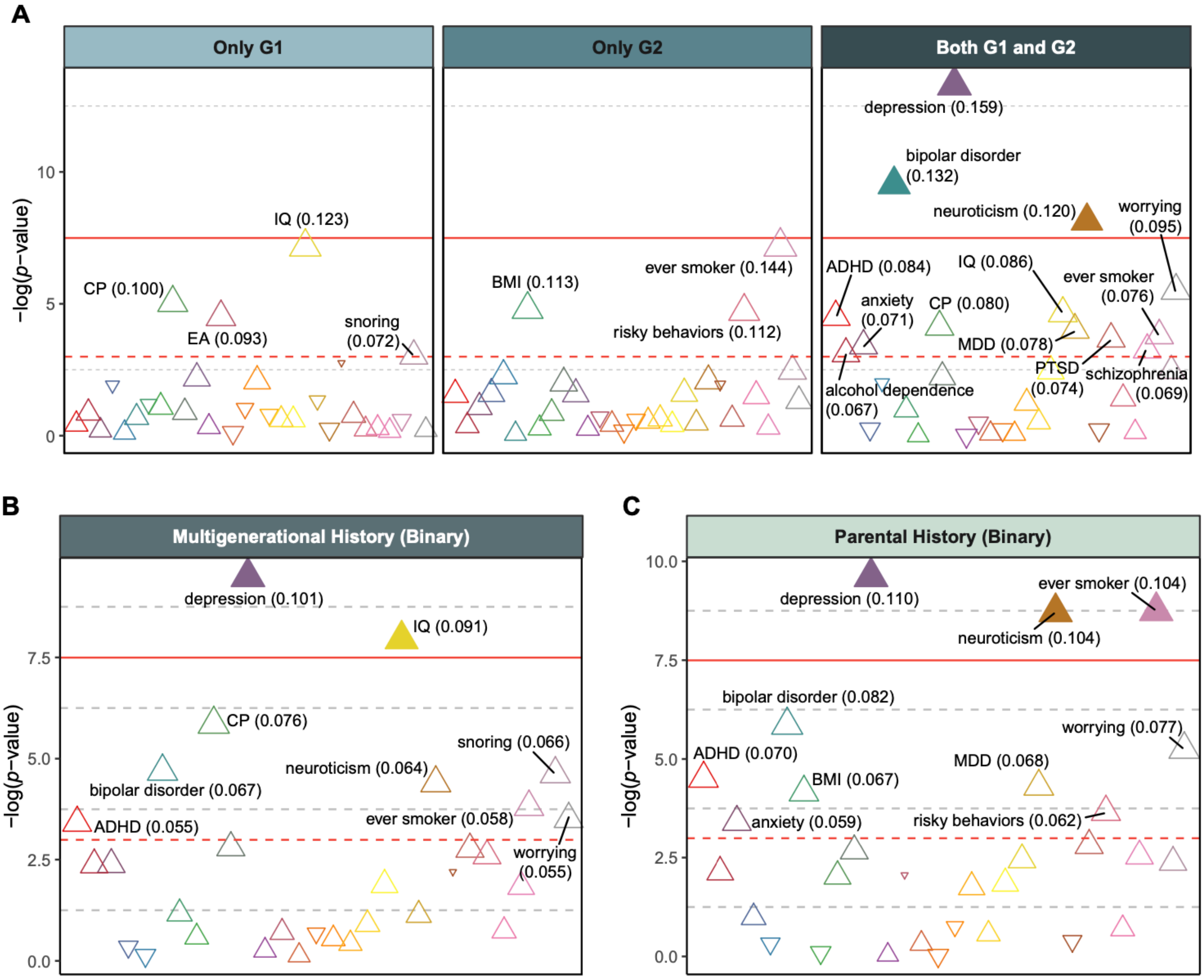

Dashed red line indicates 0.05 of unadjusted *P*. Solid red line indicates 0.05 of Bonferroni-corrected *P*. Each triangle represents a PGS with the odds ratio (OR) of family risk. Triangles filled with color denote PGSs with FDR-corrected $P<.05$. *P* values were adjusted for 30 tests. (A) Regression of four risk levels of family history from the two generations: no depression history (G1−/G2−; reference level), only grandparent (G1+/G2−), only parent (G1−/G2+), and both generations (G1+/G2+). For Depression PGS, the Z-test results were: G1+/G2− vs. G1−/G2+, Z = 0.067, *P* = 0.946; G1+/G2− vs. G1+/G2+, Z = −2.048, *P* = 0.041; and G1−/G2+ vs. G1+/G2+, Z = −1.934, *P* = 0.053. For Bipolar Disorder PGS, the results were: G1+/G2− vs. G1−/G2+, Z = 0.376, *P* = 0.707; G1+/G2− vs. G1+/G2+, Z = −2.170, *P* = 0.030; and G1−/G2+ vs. G1+/G2+, Z = −2.366, *P* = 0.018. For Neuroticism PGS, the results were: G1+/G2− vs. G1−/G2+, Z = −1.346, *P* = 0.178; G1+/G2− vs. G1+/G2+, Z = −2.642, *P* = 0.008; and G1−/G2+ vs. G1+/G2+, Z = −1.007, *P* = 0.314. (B) Regression of two-level familial risk: no depression history (G1−/G2−; reference level) and the rest of the groups (FamHist+). (C) Regression of parental depression history: no history in the parent (G2−; reference level) and depression in the parent (G2+). ADHD, attention-deficit/hyperactivity disorder; MDD, major depressive disorder; BMI, body mass index; EA, educational attainment; CP, cognitive performance; IQ, intelligence quotient; PTSD, post-traumatic stress disorder.

Supplementary Figure 3. Effects of family history of depression on residualized PGSs in European-ancestry children

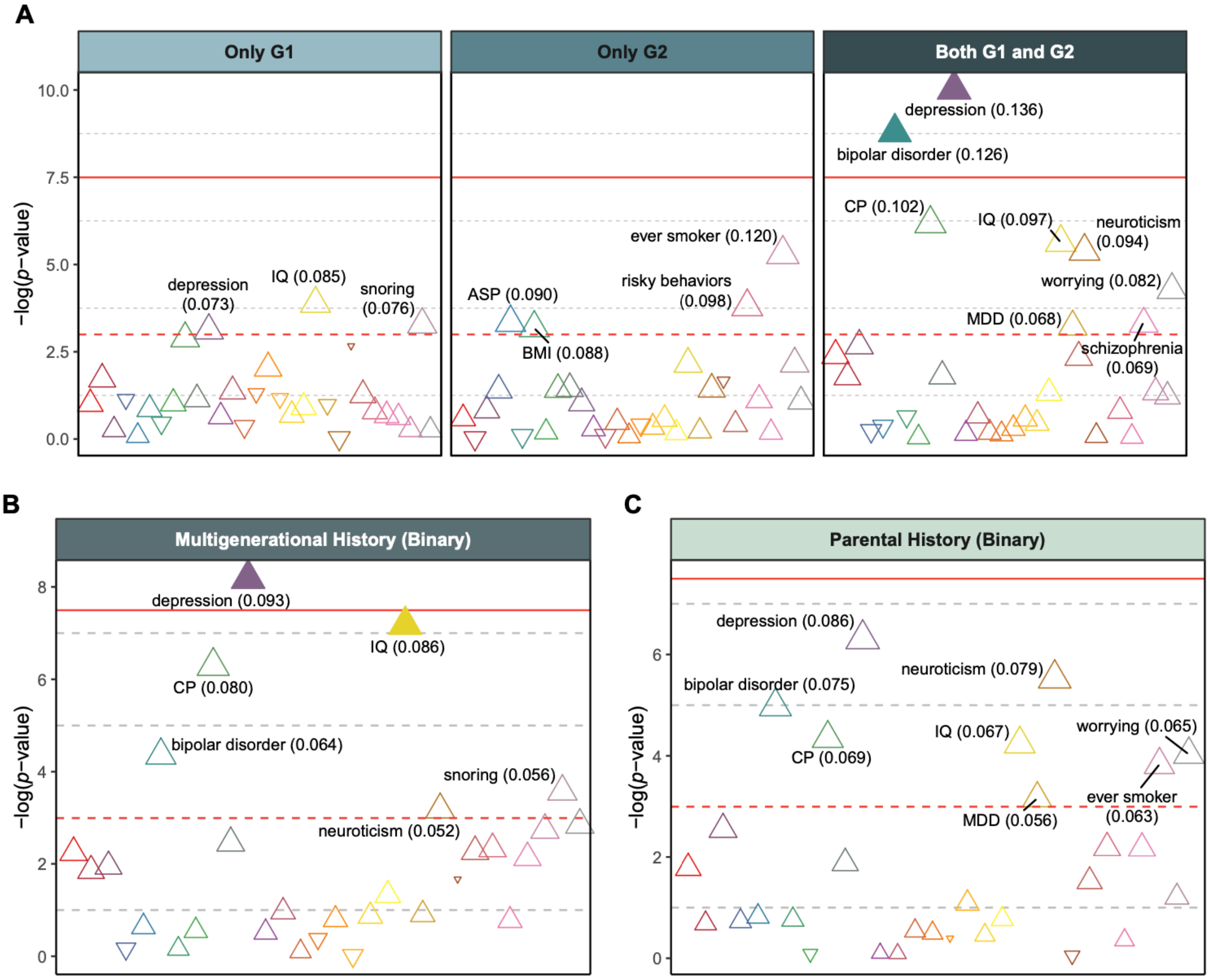

Dashed red line indicates 0.05 of unadjusted *P*. Solid red line indicates 0.05 of Bonferroni-corrected *P*. Each triangle represents a PGS with the odds ratio (OR) of family risk. Triangles filled with color denote PGSs with FDR-corrected $P<.05$. *P* values were adjusted for 30 tests. **(A)** Regression of four risk levels of family history from the two generations: no depression history (G1−/G2−; reference level), only grandparent (G1+/G2−), only parent (G1−/G2+), and both generations (G1+/G2+). Additional Z-tests were conducted to compare coefficients. For Depression PGS, the Z-test results were: G1+/G2− vs. G1−/G2+, Z = 0.604, *P* = 0.546; G1+/G2− vs. G1+/G2+, Z = −1.260, *P* = 0.208; and G1−/G2+ vs. G1+/G2+, Z = −1.774, *P* = 0.076. For Bipolar Disorder PGS, the results were: G1+/G2− vs. G1−/G2+, Z = 0.602, *P* = 0.547; G1+/G2− vs. G1+/G2+, Z = −1.958, *P* = 0.050; and G1−/G2+ vs. G1+/G2+, Z = −2.408, *P* = 0.016. **(B)** Regression of two-level familial risk: no depression history (G1−/G2−; reference level) and the rest of the groups (FamHist+). **(C)** Regression of parental depression history: no history in the parent (G2−; reference level) and depression in the parent (G2+). MDD, major depressive disorder; BMI, body mass index; ASP, automobile speed propensity; CP, cognitive performance; IQ, intelligence quotient.

Supplementary Figure 4. Effects of family history of depression on residualized PGSs in multi-ancestry children (without family history of mania)

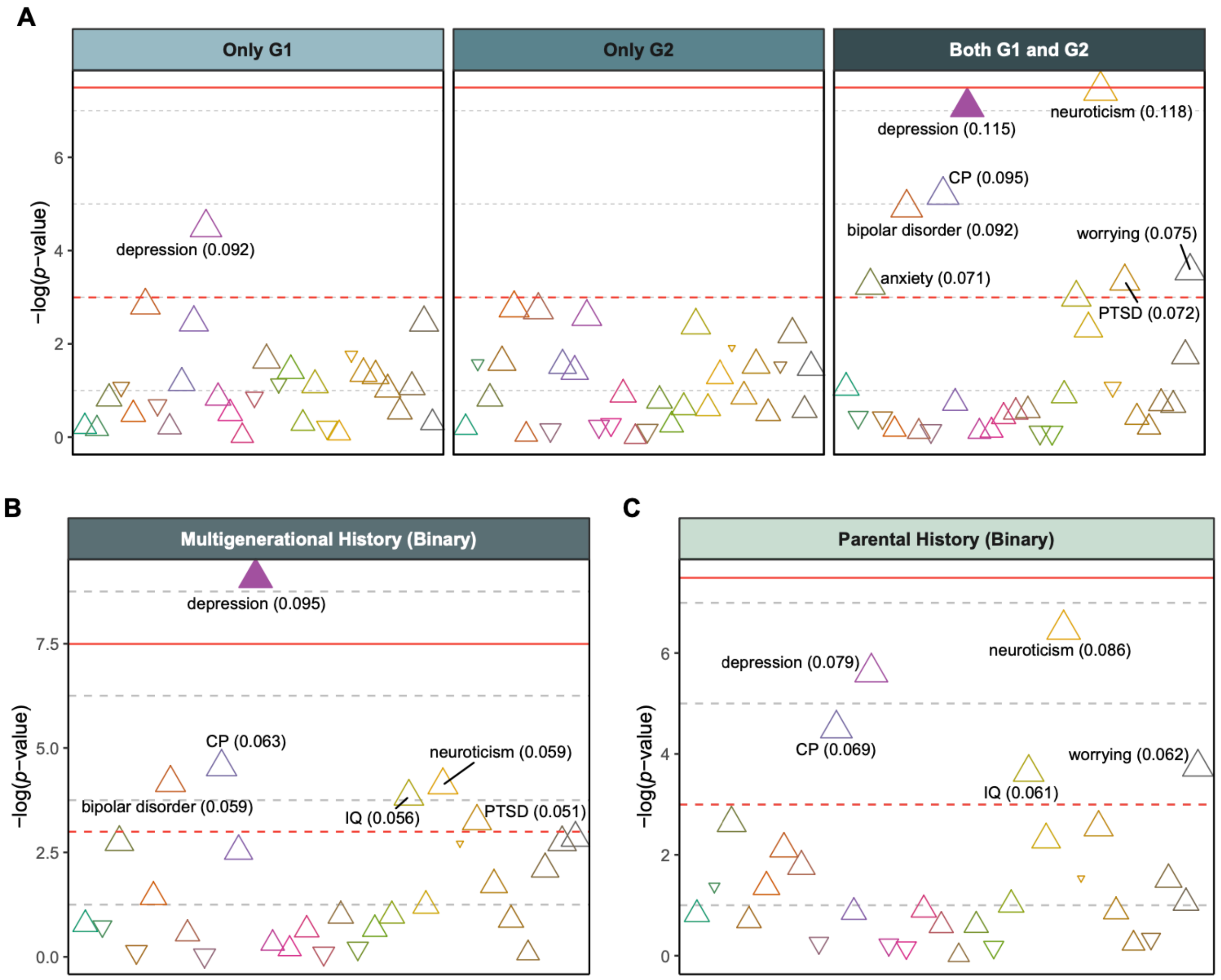

Dashed red line indicates 0.05 of unadjusted *P*. Solid red line indicates 0.05 of Bonferroni-corrected *P*. Each triangle represents a PGS with the odds ratio (OR) of family risk. Triangles filled with color denote PGSs with FDR-corrected *P*<.05. *P* values were adjusted for 30 tests. **(A)** Regression of four risk levels of family history from the two generations: no depression history (G1−/G2−; reference level), only grandparent (G1+/G2−), only parent (G1−/G2+), and both generations (G1+/G2+). **(B)** Regression of two-level familial risk: no depression history (G1−/G2−; reference level) and the rest of the groups (FamHist+). **(C)** Regression of parental depression history: no history in the parent (G2−; reference level) and depression in the parent (G2+). ADHD, attention-deficit/hyperactivity disorder; MDD, major depressive disorder; BMI, body mass index; EA, educational attainment; CP, cognitive performance; IQ, intelligence quotient; PTSD, post-traumatic stress disorder. CP, cognitive performance; IQ, intelligence quotient; PTSD, post-traumatic stress disorder.

Supplementary Figure 5. Effects of family history of depression on residualized PGSs in European-ancestry children (without family history of mania)

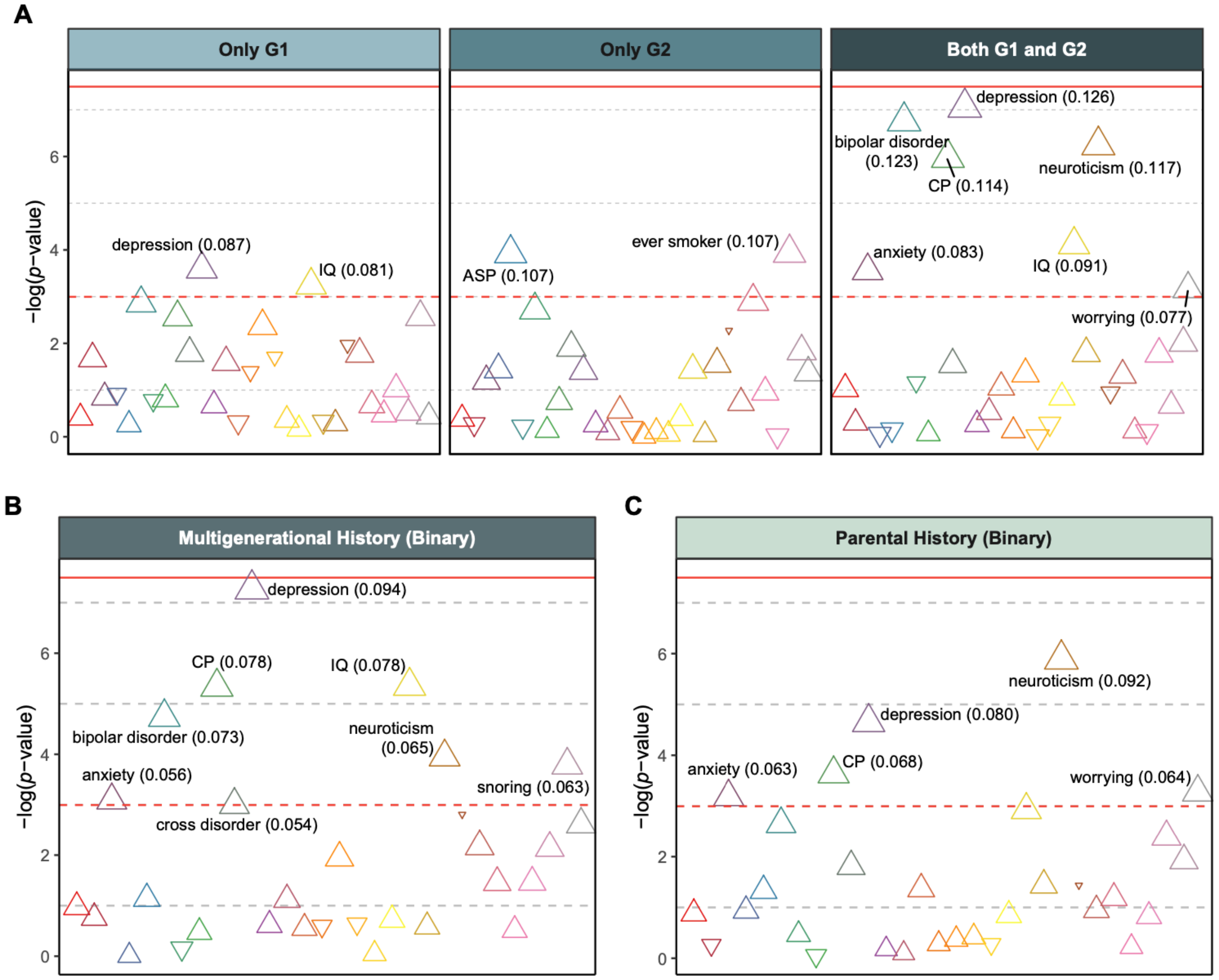

Dashed red line indicates 0.05 of unadjusted *P*. Solid red line indicates 0.05 of Bonferroni-corrected *P*. Each triangle represents a PGS with the odds ratio (OR) of family risk. Triangles filled with color denote PGSs with FDR-corrected $P<.05$. *P* values were adjusted for 30 tests. **(A)** Regression of four risk levels of family history from the two generations: no depression history (G1−/G2−; reference level), only grandparent (G1+/G2−), only parent (G1−/G2+), and both generations (G1+/G2+). **(B)** Regression of two-level familial risk: no depression history (G1−/G2−; reference level) and the rest of the groups (FamHist+). **(C)** Regression of parental depression history: no history in the parent (G2−; reference level) and depression in the parent (G2+). CP, cognitive performance; IQ, intelligence quotient; ASP, automobile speed propensity.

Supplementary Figure 6. Effects of depression PGS on clinical outcomes in European-ancestry children

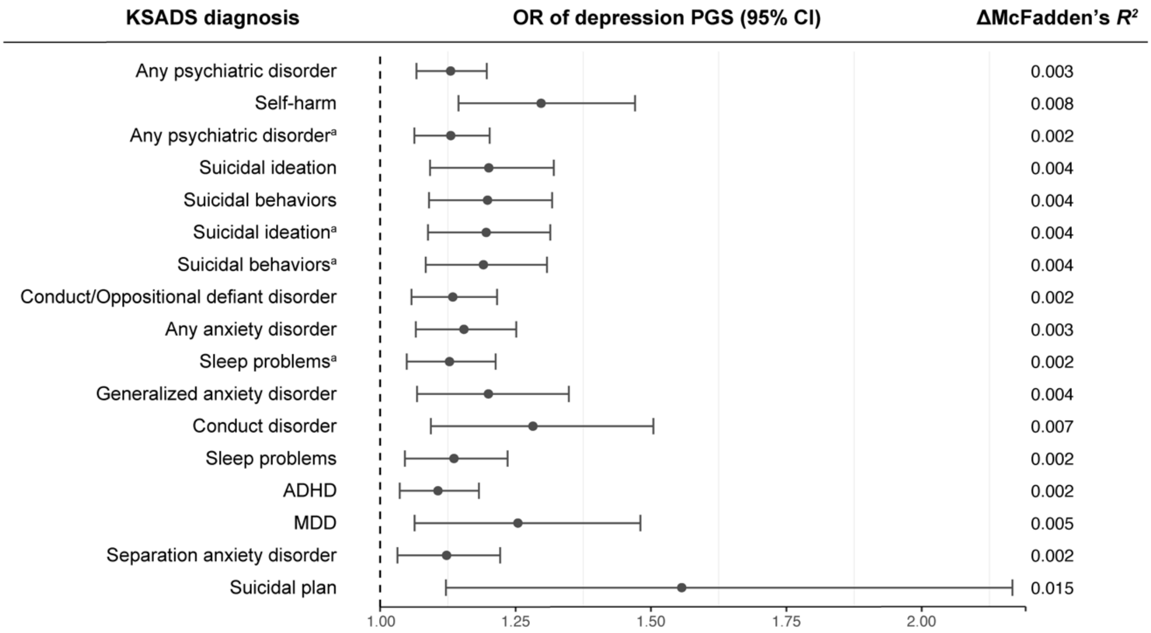

[a]reported by child; otherwise reported by parent

Presented results in the figure were from the models with significant effects of depression PGS after the FDR correction. No significant result was found with bipolar disorder PGS. Error bar indicates 95% confidence interval. *P* values were adjusted for 72 tests (36 outcomes and 2 PGSs of depression and bipolar disorder). Detailed information underlying this figure are available in Supplementary Table 6. ΔMcFadden's R^2, the proportion of variance explained by polygenic score; ADHD, attention-deficit/hyperactivity disorder; MDD, major depressive disorder.

Supplementary Figure 7. Mediation analysis with depression PGS in European-ancestry children

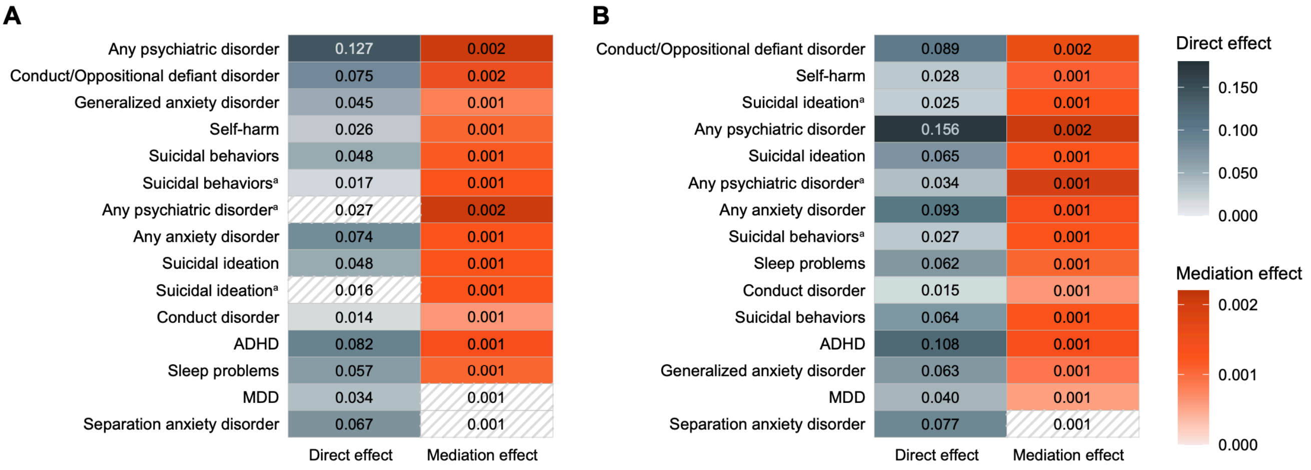

[a]reported by child; otherwise reported by parent

(A) Multigenerational family history of depression as treatment variable. (B) Parental history of depression as treatment variable. Boxes filled with color are the effects with FDR-corrected $P<.05$. P values were adjusted for 30 tests (2 versions of family history and 15 clinical outcomes). ADHD, attention-deficit/hyperactivity disorder; MDD, major depressive disorder.

Supplementary References

Auton, A., Abecasis, G. R., Altshuler, D. M., Durbin, R. M., Abecasis, G. R., Bentley, D. R., Chakravarti, A., Clark, A. G., Donnelly, P., Eichler, E. E., Flicek, P., Gabriel, S. B., Gibbs, R. A., Green, E. D., Hurles, M. E., Knoppers, B. M., Korbel, J. O., Lander, E. S., Lee, C., . . . Abecasis, G. R. (2015). A global reference for human genetic variation. *Nature*, *526*(7571), 68-74. https://doi.org/10.1038/nature15393

Conomos, M. P., Miller, M. B., & Thornton, T. A. (2015). Robust inference of population structure for ancestry prediction and correction of stratification in the presence of relatedness. *Genet Epidemiol*, *39*(4), 276-293. https://doi.org/10.1002/gepi.21896

Conomos, M. P., Reiner, A. P., Weir, B. S., & Thornton, T. A. (2016). Model-free Estimation of Recent Genetic Relatedness. *Am J Hum Genet*, *98*(1), 127-148. https://doi.org/10.1016/j.ajhg.2015.11.022

Das, S., Forer, L., Schönherr, S., Sidore, C., Locke, A. E., Kwong, A., Vrieze, S. I., Chew, E. Y., Levy, S., Mcgue, M., Schlessinger, D., Stambolian, D., Loh, P.-R., Iacono, W. G., Swaroop, A., Scott, L. J., Cucca, F., Kronenberg, F., Boehnke, M., . . . Fuchsberger, C. (2016). Next-generation genotype imputation service and methods. *Nature Genetics*, *48*(10), 1284-1287. https://doi.org/10.1038/ng.3656

Ge, T., Chen, C.-Y., Ni, Y., Feng, Y.-C. A., & Smoller, J. W. (2019). Polygenic prediction via Bayesian regression and continuous shrinkage priors. *Nature Communications*, *10*(1). https://doi.org/10.1038/s41467-019-09718-5

Loh, P.-R., Danecek, P., Palamara, P. F., Fuchsberger, C., A Reshef, Y., K Finucane, H., Schoenherr, S., Forer, L., Mccarthy, S., Abecasis, G. R., Durbin, R., & L Price, A. (2016). Reference-based phasing using the Haplotype Reference Consortium panel. *Nature Genetics*, *48*(11), 1443-1448. https://doi.org/10.1038/ng.3679

Raj, A., Stephens, M., & Pritchard, J. K. (2014). fastSTRUCTURE: variational inference of population structure in large SNP data sets. *Genetics*, *197*(2), 573-589. https://doi.org/10.1534/genetics.114.164350

Van Dijk, M. T., Murphy, E., Posner, J. E., Talati, A., & Weissman, M. M. (2021). Association of Multigenerational Family History of Depression With Lifetime Depressive and Other Psychiatric Disorders in Children. *JAMA Psychiatry*, *78*(7), 778. https://doi.org/10.1001/jamapsychiatry.2021.0350

Supplementary Information

Polygenic scores for psychiatric traits mediate the impact of multigenerational history for depression on offspring psychopathology

Eunji Lee*, Milenna T. van Dijk*, Bo-gyeom Kim, Gakyung Kim, Eleanor Murphy, Ardesheer Talati, Yoonjung Y. Joo, Myrna M. Weissman, Jiook Cha

Table of Contents

Supplementary Table 1. List of genome-wide association studies to estimate polygenic scores
Supplementary Table 2. Effects of four-level multigenerational depression risk on residualized PGS in multi-ancestry children
Supplementary Table 3. Effects of continuous multigenerational depression risk on residualized PGS in multi-ancestry children
Supplementary Table 4. Effects of continuous multigenerational depression risk on residualized PGS in European children
Supplementary Table 5. Effects of binary multigenerational depression risk on original PGS in multi-ancestry children
Supplementary Table 6. Effects of binary parental depression risk on residualized PGS in multi-ancestry children
Supplementary Table 7. Effects of depression and bipolar PGSs on KSADS diagnosis in multi-ancestry children
Supplementary Table 8. Effects of depression and bipolar PGSs on KSADS diagnosis in European-ancestry children
Supplementary Table 9. Effects of family history of depression on KSADS diagnosis in multi-ancestry children
Supplementary Table 10. Effects of family history of depression on KSADS diagnosis in European-ancestry children
Supplementary Table 11. Effects of family history of depression and depression PGS on KSADS diagnosis in multi-ancestry children
Supplementary Table 12. Effects of family history of depression and depression PGS on KSADS diagnosis in European-ancestry children

Supplementary Table 1. List of genome-wide association studies to estimate polygenic scores

Trait	Ancestry	GWAS sample size	Study
Depression	European	500,199	Howard DM, Adams MJ, Clarke T-K, et al. Genome-wide meta-analysis of depression identifies 102 independent variants and highlights the importance of the prefrontal brain regions. *Nature Neuroscience*. 2019-03-01 2019;22(3):343-352. Doi:10.1038/s41593-018-0326-7
	American	3,308	Shen H, Gelaye B, Huang H, Rondon MB, Sanchez S, Duncan LE. Polygenic prediction and GWAS of depression, PTSD, and suicidal ideation/self-harm in a Peruvian cohort. *Neuropsychopharmacology*. 2020-09-01 2020;45(10):1595-1602. Doi:10.1038/s41386-020-0603-5
Major depressive disorder (MDD)	European	480,359	Wray NR, Ripke S, Mattheisen M, et al. Genome-wide association analyses identify 44 risk variants and refine the genetic architecture of major depression. *Nature Genetics*. 2018-05-01 2018;50(5):668-681. Doi:10.1038/s41588-018-0090-3
Attention-deficit/hyperactivity disorder (ADHD)	European	53,293	Demontis D, Walters RK, Martin J, et al. Discovery of the first genome-wide significant risk loci for attention deficit/hyperactivity disorder. *Nature Genetics*. 2019-01-01 2019;51(1):63-75. Doi:10.1038/s41588-018-0269-7
General happiness	European	125,527	UK Biobank GWAS. Neale Lab. http://www.nealelab.is/ukbiobank/ Accessed Apr 29. 2020.
General happiness with own health (Happiness-health)	European	125,870	UK Biobank GWAS. Neale Lab. http://www.nealelab.is/ukbiobank/ Accessed Apr 29. 2020.
A Belief that own life is meaningful (Happiness-life)	European	123,223	UK Biobank GWAS. Neale Lab. http://www.nealelab.is/ukbiobank/ Accessed Apr 29. 2020.
Subjective well-being	European	128,049	Okbay A, Baselmans BML, De Neve J-E, et al. Genetic variants associated with subjective well-being, depressive symptoms, and neuroticism identified through genome-wide analyses. *Nature Genetics*. 2016-06-01 2016;48(6):624-633. Doi:10.1038/ng.3552
Insomnia	European	386,533	Jansen PR, Watanabe K, Stringer S, et al. Genome-wide analysis of insomnia in 1,331,010 individuals identifies new risk loci and functional pathways. *Nature Genetics*. 2019-03-01 2019;51(3):394-403. Doi:10.1038/s41588-018-0333-3
Snoring	European	359,916	Jansen PR, Watanabe K, Stringer S, et al. Genome-wide analysis of insomnia in 1,331,010 individuals identifies new risk loci and functional pathways. *Nature Genetics*. 2019-03-01 2019;51(3):394-403. Doi:10.1038/s41588-018-0333-3
Body mass index (BMI)	European	339,224	Locke AE, Kahali B, Berndt SI, et al. Genetic studies of body mass index yield new insights for obesity biology. *Nature*. 2015-02-12 2015;518(7538):197-206. Doi:10.1038/nature14177
	East Asian	173,430	Akiyama M, Okada Y, Kanai M, et al. Genome-wide association study identifies 112 new loci for body mass index in the Japanese population. *Nature Genetics*. 2017-10-01 2017;49(10):1458-1467. Doi:10.1038/ng.3951
Post-traumatic stress disorder (PTSD)	European	174,659	Nievergelt CM, Maihofer AX, Klengel T, et al. International meta-analysis of PTSD genome-wide association studies identifies sex- and ancestry-specific genetic risk loci. *Nature Communications*. 2019-12-01 2019;10(1)doi:10.1038/s41467-019-12576-w
	African	15,339	Nievergelt CM, Maihofer AX, Klengel T, et al. International meta-analysis of PTSD genome-wide association studies identifies sex- and ancestry-specific genetic risk loci. *Nature Communications*. 2019-12-01 2019;10(1)doi:10.1038/s41467-019-12576-w
	American	5,703	Nievergelt CM, Maihofer AX, Klengel T, et al. International meta-analysis of PTSD genome-wide association studies identifies sex- and ancestry-specific genetic risk loci. *Nature Communications*. 2019-12-01 2019;10(1)doi:10.1038/s41467-019-12576-w
Cognitive performance (CP)	European	1,131,881	Lee JJ, Wedow R, Okbay A, et al. Gene discovery and polygenic prediction from a genome-wide association study of educational attainment in 1.1 million individuals. *Nature Genetics*. 2018-08-01 2018;50(8):1112-1121. Doi:10.1038/s41588-018-0147-3
Educational attainment (EA)	European	1,131,881	Lee JJ, Wedow R, Okbay A, et al. Gene discovery and polygenic prediction from a genome-wide association study of educational attainment in 1.1 million individuals. *Nature Genetics*. 2018-08-01 2018;50(8):1112-1121. Doi:10.1038/s41588-018-0147-3
Intelligence quotient (IQ)	European	269,867	Savage JE, Jansen PR, Stringer S, et al. Genome-wide association meta-analysis in 269,867 individuals identifies new genetic and functional links to intelligence. *Nature Genetics*. 2018-07-01 2018;50(7):912-919. Doi:10.1038/s41588-018-0152-6

Shared effects on five major psychiatric disorders (Cross disorder)	European	61,220	Cross-Disorder Group of the Psychiatric Genomics Consortium. Identification of risk loci with shared effects on five major psychiatric disorders: a genome-wide analysis. *The Lancet*. 2013-04-01 2013;381(9875):1371-1379. Doi:10.1016/s0140-6736(12)62129-1
Neuroticism	European	390,278	Nagel M, Jansen PR, Stringer S, et al. Meta-analysis of genome-wide association studies for neuroticism in 449,484 individuals identifies novel genetic loci and pathways. *Nature Genetics*. 2018-07-01 2018;50(7):920-927. Doi:10.1038/s41588-018-0151-7
Automobile speeding propensity (ASP)	European	404,291	Karlsson Linnér R, Biroli P, Kong E, et al. Genome-wide association analyses of risk tolerance and risky behaviors in over 1 million individuals identify hundreds of loci and shared genetic influences. *Nature Genetics*. 2019-02-01 2019;51(2):245-257. Doi:10.1038/s41588-018-0309-3
The first principal components of four risky behaviors (Risky behaviors)	European	315,894	Karlsson Linnér R, Biroli P, Kong E, et al. Genome-wide association analyses of risk tolerance and risky behaviors in over 1 million individuals identify hundreds of loci and shared genetic influences. *Nature Genetics*. 2019-02-01 2019;51(2):245-257. Doi:10.1038/s41588-018-0309-3
General risk tolerance	European	466,571	Karlsson Linnér R, Biroli P, Kong E, et al. Genome-wide association analyses of risk tolerance and risky behaviors in over 1 million individuals identify hundreds of loci and shared genetic influences. *Nature Genetics*. 2019-02-01 2019;51(2):245-257. Doi:10.1038/s41588-018-0309-3
Obsessive-compulsive disorder (OCD)	European	6,518	Arnold PD, Askland KD, Barlassina C, et al. Revealing the complex genetic architecture of obsessive–compulsive disorder using meta-analysis. *Molecular Psychiatry*. 2018-05-01 2018;23(5):1181-1188. Doi:10.1038/mp.2017.154
Worrying	European	348,219	Nagel M, Jansen PR, Stringer S, et al. Meta-analysis of genome-wide association studies for neuroticism in 449,484 individuals identifies novel genetic loci and pathways. *Nature Genetics*. 2018-07-01 2018;50(7):920-927. Doi:10.1038/s41588-018-0151-7
Alcohol dependence	European	46,568	Walters RK, Polimanti R, Johnson EC, et al. Transancestral GWAS of alcohol dependence reveals common genetic underpinnings with psychiatric disorders. *Nature Neuroscience*. 2018-12-01 2018;21(12):1656-1669. Doi:10.1038/s41593-018-0275-1
	African	6,280	Walters RK, Polimanti R, Johnson EC, et al. Transancestral GWAS of alcohol dependence reveals common genetic underpinnings with psychiatric disorders. *Nature Neuroscience*. 2018-12-01 2018;21(12):1656-1669. doi:10.1038/s41593-018-0275-1
Autism spectrum disorder (ASD)	European	46,350	Grove J, Ripke S, Als TD, et al. Identification of common genetic risk variants for autism spectrum disorder. Nature Genetics. 2019-03-01 2019;51(3):431-444. doi:10.1038/s41588-019-0344-8
Alcoholic drinks consumption per week (Drinking)	European	414,343	Karlsson Linnér R, Biroli P, Kong E, et al. Genome-wide association analyses of risk tolerance and risky behaviors in over 1 million individuals identify hundreds of loci and shared genetic influences. *Nature Genetics*. 2019-02-01 2019;51(2):245-257. doi:10.1038/s41588-018-0309-3
Anorexia nervosa	European	72,517	Watson HJ, Yilmaz Z, Thornton LM, et al. Genome-wide association study identifies eight risk loci and implicates metabo-psychiatric origins for anorexia nervosa. *Nature Genetics*. 2019-08-01 2019;51(8):1207-1214. doi:10.1038/s41588-019-0439-2
Cannabis use during lifetime (Cannabis use)	European	162,082	Pasman JA, Verweij KJH, Gerring Z, et al. GWAS of lifetime cannabis use reveals new risk loci, genetic overlap with psychiatric traits, and a causal effect of schizophrenia liability. *Nature Neuroscience*. 2018-09-01 2018;21(9):1161-1170. doi:10.1038/s41593-018-0206-1
Ever been a smoker during lifetime (Ever smoker)	European	518,633	Karlsson Linnér R, Biroli P, Kong E, et al. Genome-wide association analyses of risk tolerance and risky behaviors in over 1 million individuals identify hundreds of loci and shared genetic influences. *Nature Genetics*. 2019-02-01 2019;51(2):245-257. doi:10.1038/s41588-018-0309-3
Schizophrenia	European	65,967	Ruderfer DM, Ripke S, Mcquillin A, et al. Genomic Dissection of Bipolar Disorder and Schizophrenia, Including 28 Subphenotypes. *Cell*. 2018-06-01 2018;173(7):1705-1715.e16. doi:10.1016/j.cell.2018.05.046
	East Asian	58,140	Lam M, Chen C-Y, Li Z, et al. Comparative genetic architectures of schizophrenia in East Asian and European populations. *Nature Genetics*. 2019-12-01 2019;51(12):1670-1678. doi:10.1038/s41588-019-0512-x
Bipolar disorder	European	51,710	Stahl EA, Breen G, Forstner AJ, et al. Genome-wide association study identifies 30 loci associated with bipolar disorder. *Nature Genetics*. 2019-05-01 2019;51(5):793-803. doi:10.1038/s41588-019-0397-8
Anxiety	European	31,060	Otowa T, Hek K, Lee M, et al. Meta-analysis of genome-wide association studies of anxiety disorders. *Molecular Psychiatry*. 2016-10-01 2016;21(10):1391-1399. doi:10.1038/mp.2015.197

Supplementary Table 2. Effects of four-level multigenerational depression risk on residualized PGS in multi-ancestry children

PGS	Estimate [95% Confidence interval]			Unadjusted *P* value			FDR-corrected *P*<.05			Bonferroni-corrected *P*<.05		
	G1+/G2-	G1-/G2+	G1+/G2+	G1+/G2-	G1-/G2+	G1+/G2+	G1+/G2-	G1-/G2+	G1+/G2+	G1+/G2-	G1-/G2+	G1+/G2+
depression	0.079 [0.013, 0.145]	0.057 [-0.015, 0.128]	0.129 [0.070, 0.187]	0.019	0.123	1.55E-05	FALSE	FALSE	TRUE	FALSE	FALSE	TRUE
bipolar disorder	0.034 [-0.032, 0.100]	0.007 [-0.065, 0.079]	0.109 [0.051, 0.168]	0.312	0.847	2.53E-04	FALSE	FALSE	TRUE	FALSE	FALSE	TRUE
neuroticism	-0.014 [-0.080, 0.052]	0.041 [-0.031, 0.113]	0.093 [0.035, 0.152]	0.681	0.264	0.002	FALSE	FALSE	FALSE	FALSE	FALSE	FALSE
CP	0.034 [-0.032, 0.100]	0.052 [-0.020, 0.124]	0.077 [0.018, 0.135]	0.316	0.155	0.01	FALSE	FALSE	FALSE	FALSE	FALSE	FALSE
worrying	0.002 [-0.064, 0.068]	0.044 [-0.028, 0.116]	0.076 [0.018, 0.135]	0.95	0.228	0.01	FALSE	FALSE	FALSE	FALSE	FALSE	FALSE
MDD	-0.020 [-0.086, 0.046]	0.022 [-0.050, 0.094]	0.070 [0.011, 0.128]	0.558	0.545	0.019	FALSE	FALSE	FALSE	FALSE	FALSE	FALSE
IQ	0.037 [-0.029, 0.102]	0.067 [-0.005, 0.139]	0.065 [0.007, 0.124]	0.277	0.07	0.029	FALSE	FALSE	FALSE	FALSE	FALSE	FALSE
PTSD	0.032 [-0.034, 0.098]	0.022 [-0.050, 0.094]	0.064 [0.006, 0.123]	0.347	0.545	0.031	FALSE	FALSE	FALSE	FALSE	FALSE	FALSE
ADHD	0.020 [-0.046, 0.086]	0.009 [-0.063, 0.081]	0.063 [0.004, 0.121]	0.556	0.802	0.036	FALSE	FALSE	FALSE	FALSE	FALSE	FALSE
schizophrenia	0.013 [-0.053, 0.079]	-0.030 [-0.102, 0.042]	0.057 [-0.002, 0.115]	0.698	0.415	0.058	FALSE	FALSE	FALSE	FALSE	FALSE	FALSE
anxiety	0.013 [-0.053, 0.079]	0.029 [-0.043, 0.101]	0.052 [-0.007, 0.110]	0.707	0.437	0.084	FALSE	FALSE	FALSE	FALSE	FALSE	FALSE
ever smoker	0.028 [-0.038, 0.094]	0.078 [0.006, 0.150]	0.045 [-0.014, 0.103]	0.403	0.034	0.132	FALSE	FALSE	FALSE	FALSE	FALSE	FALSE
insomnia	0.026 [-0.040, 0.092]	0.013 [-0.059, 0.085]	0.038 [-0.021, 0.096]	0.434	0.714	0.207	FALSE	FALSE	FALSE	FALSE	FALSE	FALSE
snoring	0.062 [-0.004, 0.128]	0.026 [-0.046, 0.098]	0.036 [-0.022, 0.095]	0.064	0.481	0.223	FALSE	FALSE	FALSE	FALSE	FALSE	FALSE
risky behaviors	0.039 [-0.027, 0.105]	0.054 [-0.018, 0.126]	0.036 [-0.023, 0.094]	0.249	0.139	0.233	FALSE	FALSE	FALSE	FALSE	FALSE	FALSE
cross disorder	0.051 [-0.015, 0.117]	0.032 [-0.040, 0.104]	0.034 [-0.024, 0.093]	0.131	0.384	0.25	FALSE	FALSE	FALSE	FALSE	FALSE	FALSE
alcohol dependence	0.025 [-0.041, 0.091]	-0.011 [-0.083, 0.061]	0.024 [-0.035, 0.082]	0.46	0.773	0.428	FALSE	FALSE	FALSE	FALSE	FALSE	FALSE
SWB	0.044 [-0.022, 0.110]	0.035 [-0.037, 0.107]	0.023 [-0.036, 0.081]	0.195	0.343	0.449	FALSE	FALSE	FALSE	FALSE	FALSE	FALSE
BMI	-0.021 [-0.087, 0.045]	0.086 [0.014, 0.158]	0.022 [-0.036, 0.081]	0.526	0.02	0.457	FALSE	FALSE	FALSE	FALSE	FALSE	FALSE
general happiness	-0.023 [-0.089, 0.043]	0.013 [-0.059, 0.085]	0.021 [-0.037, 0.080]	0.5	0.718	0.472	FALSE	FALSE	FALSE	FALSE	FALSE	FALSE
risk tolerance	0.032 [-0.034, 0.098]	0.025 [-0.047, 0.097]	0.017 [-0.041, 0.076]	0.337	0.502	0.568	FALSE	FALSE	FALSE	FALSE	FALSE	FALSE
happiness-life	-0.017 [-0.083, 0.049]	0.022 [-0.050, 0.094]	0.016 [-0.042, 0.075]	0.611	0.546	0.588	FALSE	FALSE	FALSE	FALSE	FALSE	FALSE
ASD	-0.034 [-0.100, 0.032]	0.032 [-0.040, 0.104]	-0.016 [-0.074, 0.043]	0.307	0.379	0.602	FALSE	FALSE	FALSE	FALSE	FALSE	FALSE
happiness-health	0.040 [-0.026, 0.106]	-0.012 [-0.084, 0.060]	-0.005 [-0.064, 0.053]	0.23	0.742	0.862	FALSE	FALSE	FALSE	FALSE	FALSE	FALSE
cannabis use	0.013 [-0.053, 0.079]	-0.008 [-0.080, 0.064]	-0.004 [-0.062, 0.055]	0.696	0.822	0.899	FALSE	FALSE	FALSE	FALSE	FALSE	FALSE
OCD	-0.045 [-0.111, 0.021]	-0.044 [-0.116, 0.028]	-0.003 [-0.062, 0.055]	0.177	0.229	0.914	FALSE	FALSE	FALSE	FALSE	FALSE	FALSE
ASP	0.009 [-0.057, 0.075]	0.067 [-0.005, 0.139]	0.002 [-0.056, 0.061]	0.793	0.07	0.936	FALSE	FALSE	FALSE	FALSE	FALSE	FALSE
drinking	0.029 [-0.036, 0.095]	-0.017 [-0.089, 0.055]	-0.002 [-0.060, 0.057]	0.381	0.635	0.958	FALSE	FALSE	FALSE	FALSE	FALSE	FALSE
EA	0.014 [-0.052, 0.080]	-0.026 [-0.098, 0.046]	-0.001 [-0.060, 0.057]	0.671	0.479	0.962	FALSE	FALSE	FALSE	FALSE	FALSE	FALSE
anorexia nervosa	-0.002 [-0.068, 0.064]	0.021 [-0.051, 0.093]	0.001 [-0.057, 0.060]	0.951	0.571	0.963	FALSE	FALSE	FALSE	FALSE	FALSE	FALSE

Regression of four risk levels of family history from the two generations: no depression history (G1−/G2−; reference level), only grandparent (G1+/G2−), only parent (G1−/G2+), and both generations (G1+/G2+). *P* values were adjusted for 30 tests. Additional Z-tests were conducted to compare coefficients. For Depression PGS, the Z-test results were as follows: G1+/G2- vs. G1-/G2+, Z = 0.446, *P* = 0.656; G1+/G2- vs. G1+/G2+, Z = -1.117, *P* = 0.264; and G1-/G2+ vs. G1+/G2+, Z = -1.531, *P* = 0.126. For Bipolar Disorder PGS, the Z-test results were: G1+/G2- vs. G1-/G2+, Z = 0.541, *P* = 0.588; G1+/G2- vs. G1+/G2+, Z = -1.673, *P* = 0.094; and G1-/G2+ vs. G1+/G2+, Z = -2.159, *P* = 0.031.

Supplementary Table 3. Effects of continuous multigenerational depression risk on residualized PGS in multi-ancestry children

PGS	Estimate [95% Confidence interval]	Unadjusted *P* value	FDR-corrected *P*<.05	Bonferroni-corrected *P*<.05
depression	0.041 [0.022, 0.059]	1.34E-05	TRUE	TRUE
bipolar disorder	0.031 [0.012, 0.049]	0.001	TRUE	FALSE
neuroticism	0.029 [0.011, 0.047]	0.002	FALSE	FALSE
CP	0.026 [0.007, 0.044]	0.006	FALSE	FALSE
worrying	0.025 [0.006, 0.043]	0.009	FALSE	FALSE
IQ	0.024 [0.005, 0.042]	0.011	FALSE	FALSE
MDD	0.021 [0.002, 0.039]	0.027	FALSE	FALSE
PTSD	0.020 [0.001, 0.038]	0.035	FALSE	FALSE
ever smoker	0.019 [0.001, 0.038]	0.041	FALSE	FALSE
ADHD	0.018 [-2.659734e-04, 0.036]	0.053	FALSE	FALSE
anxiety	0.017 [-0.002, 0.035]	0.076	FALSE	FALSE
risky behaviors	0.015 [-0.004, 0.033]	0.116	FALSE	FALSE
BMI	0.013 [-0.005, 0.032]	0.16	FALSE	FALSE
schizophrenia	0.013 [-0.005, 0.031]	0.164	FALSE	FALSE
snoring	0.013 [-0.006, 0.031]	0.174	FALSE	FALSE
cross disorder	0.013 [-0.006, 0.031]	0.179	FALSE	FALSE
insomnia	0.012 [-0.007, 0.030]	0.212	FALSE	FALSE
SWB	0.010 [-0.009, 0.028]	0.308	FALSE	FALSE
risk tolerance	0.007 [-0.011, 0.025]	0.451	FALSE	FALSE
general happiness	0.007 [-0.012, 0.025]	0.47	FALSE	FALSE
ASP	0.006 [-0.012, 0.025]	0.493	FALSE	FALSE
happiness-life	0.006 [-0.012, 0.025]	0.512	FALSE	FALSE
alcohol dependence	0.006 [-0.013, 0.024]	0.537	FALSE	FALSE
OCD	-0.005 [-0.023, 0.013]	0.587	FALSE	FALSE
EA	-0.002 [-0.021, 0.016]	0.792	FALSE	FALSE
eating disorder	0.002 [-0.016, 0.021]	0.82	FALSE	FALSE
happiness-health	-0.002 [-0.020, 0.016]	0.826	FALSE	FALSE
ASD	-0.002 [-0.020, 0.017]	0.845	FALSE	FALSE
drinking	-0.002 [-0.020, 0.017]	0.861	FALSE	FALSE
cannabis	-0.002 [-0.020, 0.017]	0.864	FALSE	FALSE

P values were adjusted for 30 tests.

Supplementary Table 4. Effects of continuous multigenerational depression risk on residualized PGS in European children

PGS	Estimate [95% Confidence interval]	Unadjusted *P* value	FDR-corrected *P*<.05	Bonferroni-corrected *P*<.05
depression	0.041 [0.021, 0.062]	8.81E-05	TRUE	TRUE
bipolar disorder	0.035 [0.015, 0.056]	0.001	TRUE	FALSE
CP	0.033 [0.012, 0.054]	0.002	FALSE	FALSE
IQ	0.033 [0.012, 0.053]	0.002	TRUE	FALSE
neuroticism	0.030 [0.010, 0.051]	0.004	FALSE	FALSE
worrying	0.026 [0.005, 0.047]	0.013	FALSE	FALSE
schizophrenia	0.020 [-3.736709e-04, 0.041]	0.054	FALSE	FALSE
MDD	0.020 [-0.001, 0.041]	0.058	FALSE	FALSE
anxiety	0.020 [-0.001, 0.040]	0.064	FALSE	FALSE
ever smoker	0.020 [-0.001, 0.040]	0.065	FALSE	FALSE
ADHD	0.018 [-0.003, 0.039]	0.092	FALSE	FALSE
PTSD	0.017 [-0.004, 0.038]	0.105	FALSE	FALSE
cross disorder	0.017 [-0.004, 0.038]	0.11	FALSE	FALSE
snoring	0.015 [-0.006, 0.036]	0.155	FALSE	FALSE
risky behaviors	0.014 [-0.006, 0.035]	0.172	FALSE	FALSE
alcohol dependence	0.013 [-0.008, 0.034]	0.221	FALSE	FALSE
insomnia	0.011 [-0.010, 0.032]	0.292	FALSE	FALSE
happiness-life	0.007 [-0.014, 0.027]	0.538	FALSE	FALSE
SWB	0.006 [-0.014, 0.027]	0.552	FALSE	FALSE
EA	0.006 [-0.015, 0.027]	0.58	FALSE	FALSE
risk tolerance	0.004 [-0.017, 0.025]	0.71	FALSE	FALSE
eating disorder	0.004 [-0.017, 0.024]	0.738	FALSE	FALSE
OCD	-0.003 [-0.024, 0.017]	0.753	FALSE	FALSE
ASP	0.003 [-0.018, 0.024]	0.777	FALSE	FALSE
drinking	0.003 [-0.018, 0.023]	0.795	FALSE	FALSE
happiness-health	0.002 [-0.019, 0.023]	0.861	FALSE	FALSE
general happiness	0.002 [-0.019, 0.022]	0.87	FALSE	FALSE
cannabis use	0.001 [-0.019, 0.022]	0.898	FALSE	FALSE
ASD	0.001 [-0.020, 0.022]	0.918	FALSE	FALSE
BMI	4.974e-04 [-0.020, 0.021]	0.963	FALSE	FALSE

P values were adjusted for 30 tests.

Supplementary Table 5. Effects of binary multigenerational depression risk on original PGS in multi-ancestry children

PGS	Estimate [95% Confidence interval]	Unadjusted *P* value	FDR-corrected *P*<.05	Bonferroni-corrected *P*<.05
depression	0.095 [0.051, 0.139]	2.54E-05	TRUE	TRUE
bipolar disorder	0.059 [0.015, 0.103]	0.008	FALSE	FALSE
CP	0.057 [0.013, 0.101]	0.011	FALSE	FALSE
IQ	0.056 [0.012, 0.100]	0.012	FALSE	FALSE
ever smoker	0.048 [0.004, 0.092]	0.033	FALSE	FALSE
neuroticism	0.046 [0.002, 0.090]	0.04	FALSE	FALSE
worrying	0.045 [0.001, 0.089]	0.047	FALSE	FALSE
PTSD	0.043 [-0.001, 0.087]	0.054	FALSE	FALSE
snoring	0.042 [-0.002, 0.086]	0.062	FALSE	FALSE
risky behaviors	0.041 [-0.003, 0.085]	0.065	FALSE	FALSE
cross disorder	0.039 [-0.005, 0.083]	0.083	FALSE	FALSE
ADHD	0.035 [-0.009, 0.079]	0.114	FALSE	FALSE
anxiety	0.033 [-0.011, 0.077]	0.137	FALSE	FALSE
SWB	0.032 [-0.012, 0.076]	0.15	FALSE	FALSE
MDD	0.029 [-0.015, 0.073]	0.191	FALSE	FALSE
insomnia	0.028 [-0.016, 0.072]	0.214	FALSE	FALSE
OCD	-0.027 [-0.071, 0.017]	0.229	FALSE	FALSE
BMI	0.025 [-0.019, 0.069]	0.272	FALSE	FALSE
risk tolerance	0.024 [-0.020, 0.068]	0.289	FALSE	FALSE
ASP	0.021 [-0.023, 0.065]	0.353	FALSE	FALSE
schizophrenia	0.021 [-0.023, 0.065]	0.356	FALSE	FALSE
alcohol dependence	0.015 [-0.029, 0.059]	0.497	FALSE	FALSE
ASD	-0.009 [-0.053, 0.035]	0.679	FALSE	FALSE
happiness-health	0.007 [-0.037, 0.051]	0.74	FALSE	FALSE
happiness-life	0.007 [-0.037, 0.051]	0.749	FALSE	FALSE
general happiness	0.005 [-0.039, 0.049]	0.809	FALSE	FALSE
anorexia nervosa	0.005 [-0.039, 0.049]	0.815	FALSE	FALSE
drinking	0.004 [-0.040, 0.048]	0.852	FALSE	FALSE
EA	-0.003 [-0.047, 0.041]	0.903	FALSE	FALSE
cannabis use	4.29E-04 [-0.044, 0.044]	0.985	FALSE	FALSE

P values were adjusted for 30 tests.

Supplementary Table 6. Effects of binary parental depression risk on original PGS in multi-ancestry children

PGS	Estimate [95% Confidence interval]	Unadjusted *P* value	FDR-corrected *P*<.05	Bonferroni-corrected *P*<.05
depression	0.087 [0.039, 0.135]	3.76E-04	TRUE	TRUE
neuroticism	0.077 [0.029, 0.124]	0.002	FALSE	FALSE
bipolar disorder	0.065 [0.017, 0.112]	0.008	FALSE	FALSE
worrying	0.064 [0.016, 0.112]	0.009	FALSE	FALSE
CP	0.061 [0.013, 0.109]	0.012	FALSE	FALSE
IQ	0.059 [0.011, 0.107]	0.016	FALSE	FALSE
MDD	0.056 [0.008, 0.104]	0.022	FALSE	FALSE
ever smoker	0.052 [0.004, 0.100]	0.034	FALSE	FALSE
BMI	0.050 [0.002, 0.098]	0.041	FALSE	FALSE
PTSD	0.043 [-0.005, 0.091]	0.081	FALSE	FALSE
anxiety	0.041 [-0.007, 0.088]	0.097	FALSE	FALSE
ADHD	0.039 [-0.009, 0.087]	0.111	FALSE	FALSE
risky behaviors	0.035 [-0.013, 0.083]	0.149	FALSE	FALSE
ASP	0.025 [-0.023, 0.073]	0.312	FALSE	FALSE
cross disorder	0.024 [-0.024, 0.072]	0.33	FALSE	FALSE
insomnia	0.024 [-0.024, 0.072]	0.334	FALSE	FALSE
general happiness	0.023 [-0.025, 0.071]	0.353	FALSE	FALSE
schizophrenia	0.022 [-0.026, 0.070]	0.372	FALSE	FALSE
happiness-life	0.022 [-0.026, 0.070]	0.376	FALSE	FALSE
snoring	0.021 [-0.027, 0.069]	0.399	FALSE	FALSE
SWB	0.019 [-0.029, 0.067]	0.44	FALSE	FALSE
happiness-health	-0.015 [-0.063, 0.032]	0.528	FALSE	FALSE
risk tolerance	0.014 [-0.034, 0.062]	0.574	FALSE	FALSE
EA	-0.013 [-0.061, 0.035]	0.585	FALSE	FALSE
drinking	-0.013 [-0.061, 0.035]	0.592	FALSE	FALSE
OCD	-0.010 [-0.058, 0.038]	0.685	FALSE	FALSE
anorexia nervosa	0.009 [-0.039, 0.057]	0.712	FALSE	FALSE
ASD	0.009 [-0.039, 0.057]	0.718	FALSE	FALSE
cannabis use	-0.008 [-0.056, 0.040]	0.745	FALSE	FALSE
alcohol dependence	0.006 [-0.042, 0.054]	0.802	FALSE	FALSE

P values were adjusted for 30 tests.

Supplementary Table 7. Effects of depression and bipolar PGSs on KSADS diagnosis in multi-ancestry children

KSADS diagnosis	PGS	OR [95% Confidence interval]	Unadjusted *P* value	FDR-corrected *P*<.05	Bonferroni-corrected *P*<.05	McFadden's R^2	∆McFadden's R^2
Any psychiatric disorder	depression	1.13 [1.075, 1.188]	1.39E-06	TRUE	TRUE	0.022	0.003
Conduct/Oppositional defiant disorder	depression	1.148 [1.079, 1.221]	1.18E-05	TRUE	TRUE	0.04	0.003
Any psychiatric disorder (child)	depression	1.116 [1.059, 1.176]	3.83E-05	TRUE	TRUE	0.021	0.002
Any anxiety disorder	depression	1.153 [1.073, 1.238]	9.75E-05	TRUE	TRUE	0.027	0.003
Conduct disorder	depression	1.28 [1.120, 1.462]	2.81E-04	TRUE	TRUE	0.096	0.007
ADHD	depression	1.111 [1.049, 1.177]	3.47E-05	TRUE	TRUE	0.044	0.002
Separation anxiety disorder	depression	1.145 [1.062, 1.234]	4.34E-04	TRUE	TRUE	0.03	0.003
Suicidal ideation (child)	depression	1.146 [1.058, 1.243]	0.001	TRUE	FALSE	0.033	0.003
Self-harm	depression	1.205 [1.077, 1.347]	0.001	TRUE	FALSE	0.036	0.004
Sleep problems (child)	depression	1.106 [1.039, 1.176]	0.002	TRUE	FALSE	0.012	0.002
Generalized anxiety disorder	depression	1.187 [1.068, 1.319]	0.002	TRUE	FALSE	0.041	0.004
Suicidal behaviors (child)	depression	1.135 [1.048, 1.229]	0.002	TRUE	FALSE	0.033	0.002
Suicidal ideation	depression	1.141 [1.050, 1.240]	0.002	TRUE	FALSE	0.047	0.002
Sleep problems	depression	1.123 [1.043, 1.209]	0.002	TRUE	FALSE	0.023	0.002
Suicidal behaviors	depression	1.137 [1.046, 1.235]	0.003	TRUE	FALSE	0.046	0.002
Psychotic disorder	depression	1.193 [1.035, 1.374]	0.015	FALSE	FALSE	0.049	0.003
Any depressive disorder (child)	depression	1.135 [1.021, 1.260]	0.019	FALSE	FALSE	0.036	0.002
Simple/specific phobia	depression	1.058 [1.007, 1.111]	0.026	FALSE	FALSE	0.01	0.001
Bipolar disorder	depression	1.104 [1.010, 1.206]	0.029	FALSE	FALSE	0.057	0.001
PTSD	depression	1.187 [1.011, 1.394]	0.037	FALSE	FALSE	0.101	0.003
Any depressive disorder	depression	1.097 [1.003, 1.200]	0.042	FALSE	FALSE	0.038	0.001
Suicidal attempt (child)	bipolar disorder	1.215 [0.997, 1.479]	0.053	FALSE	FALSE	0.085	0.004
Social anxiety disorder	bipolar disorder	0.905 [0.816, 1.003]	0.057	FALSE	FALSE	0.025	0.001
Social anxiety disorder	depression	1.1 [0.991, 1.220]	0.072	FALSE	FALSE	0.025	0.001
Sleep problems (child)	bipolar disorder	0.948 [0.892, 1.008]	0.089	FALSE	FALSE	0.011	4.38E-04
MDD (child)	depression	1.127 [0.979, 1.297]	0.096	FALSE	FALSE	0.051	0.002
PTSD	bipolar disorder	1.142 [0.975, 1.338]	0.1	FALSE	FALSE	0.1	0.002
MDD	depression	1.114 [0.972, 1.278]	0.122	FALSE	FALSE	0.067	0.001
Any anxiety disorder (child)	bipolar disorder	1.1 [0.972, 1.245]	0.132	FALSE	FALSE	0.045	0.001
Suicidal attepmt	bipolar disorder	1.288 [0.924, 1.798]	0.135	FALSE	FALSE	0.106	0.006
Eating disorder	depression	0.95 [0.883, 1.022]	0.165	FALSE	FALSE	0.007	3.81E-04
Any anxiety disorder (child)	depression	1.086 [0.958, 1.230]	0.198	FALSE	FALSE	0.044	0.001
Suicidal plan	bipolar disorder	0.847 [0.657, 1.092]	0.201	FALSE	FALSE	0.129	0.003
Any anxiety disorder	bipolar disorder	0.956 [0.891, 1.026]	0.215	FALSE	FALSE	0.024	3.17E-04
MDD (child)	bipolar disorder	1.093 [0.949, 1.258]	0.218	FALSE	FALSE	0.051	0.001
Agoraphobia	bipolar disorder	1.174 [0.875, 1.575]	0.285	FALSE	FALSE	0.078	0.003
Any depressive disorder (child)	bipolar disorder	1.057 [0.951, 1.174]	0.307	FALSE	FALSE	0.034	3.94E-04
Sleep problems	bipolar disorder	0.964 [0.896, 1.037]	0.327	FALSE	FALSE	0.022	2.17E-04
Any psychiatric disorder	bipolar disorder	0.976 [0.929, 1.025]	0.33	FALSE	FALSE	0.02	1.14E-04
Panic disorder	bipolar disorder	1.22 [0.816, 1.817]	0.331	FALSE	FALSE	0.179	0.005
Panic disorder	depression	0.804 [0.526, 1.225]	0.332	FALSE	FALSE	0.179	0.005
Suicidal plan (child)	bipolar disorder	0.912 [0.749, 1.110]	0.358	FALSE	FALSE	0.072	0.001
Suicidal attempt (child)	depression	1.097 [0.896, 1.343]	0.368	FALSE	FALSE	0.082	0.001
OCD	depression	1.033 [0.957, 1.115]	0.401	FALSE	FALSE	0.031	1.74E-04
Suicidal plan	depression	1.114 [0.862, 1.440]	0.408	FALSE	FALSE	0.128	0.002

Simple/specific phobia	bipolar disorder	0.98<br>[0.933, 1.029]	0.42	FALSE	FALSE	0.01	7.51E-05
Agoraphobia	depression	1.124<br>[0.836, 1.509]	0.437	FALSE	FALSE	0.077	0.002
OCD	bipolar disorder	0.971<br>[0.900, 1.047]	0.444	FALSE	FALSE	0.031	1.53E-04
Self-harm	bipolar disorder	1.043<br>[0.934, 1.165]	0.451	FALSE	FALSE	0.032	2.84E-04
Suicidal plan (child)	depression	1.072<br>[0.880, 1.306]	0.487	FALSE	FALSE	0.071	0.001
Eating disorder	bipolar disorder	1.025<br>[0.953, 1.102]	0.511	FALSE	FALSE	0.007	9.16E-05
Any psychiatric disorder (child)	bipolar disorder	0.983<br>[0.933, 1.035]	0.514	FALSE	FALSE	0.019	6.15E-05
Conduct/Oppositional defiant disorder	bipolar disorder	0.98<br>[0.922, 1.042]	0.517	FALSE	FALSE	0.037	9.53E-05
Social anxiety disorder (child)	depression	1.084<br>[0.847, 1.387]	0.522	FALSE	FALSE	0.09	0.001
Bipolar disorder	bipolar disorder	0.973<br>[0.891, 1.062]	0.538	FALSE	FALSE	0.056	1.62E-04
Conduct disorder	bipolar disorder	0.961<br>[0.840, 1.098]	0.556	FALSE	FALSE	0.09	3.63E-04
Suicidal ideation (child)	bipolar disorder	1.024<br>[0.945, 1.110]	0.561	FALSE	FALSE	0.03	1.10E-04
Suicidal behaviors (child)	bipolar disorder	1.024<br>[0.945, 1.108]	0.567	FALSE	FALSE	0.031	1.07E-04
Bipolar disorder (child)	bipolar disorder	0.933<br>[0.735, 1.184]	0.57	FALSE	FALSE	0.072	0.001
Separation anxiety disorder	bipolar disorder	0.979<br>[0.909, 1.055]	0.582	FALSE	FALSE	0.028	9.74E-05
Generalized anxiety disorder (child)	depression	1.072<br>[0.833, 1.380]	0.589	FALSE	FALSE	0.099	0.001
Generalized anxiety disorder	bipolar disorder	1.027<br>[0.925, 1.140]	0.623	FALSE	FALSE	0.038	1.60E-04
Suicidal attepmt	depression	1.082<br>[0.776, 1.507]	0.643	FALSE	FALSE	0.101	0.001
Generalized anxiety disorder (child)	bipolar disorder	1.045<br>[0.814, 1.341]	0.729	FALSE	FALSE	0.099	0.001
Psychotic disorder	bipolar disorder	1.025<br>[0.889, 1.181]	0.733	FALSE	FALSE	0.046	1.63E-04
Bipolar disorder (child)	depression	1.042<br>[0.823, 1.320]	0.733	FALSE	FALSE	0.072	4.38E-04
Social anxiety disorder (child)	bipolar disorder	1.042<br>[0.819, 1.324]	0.74	FALSE	FALSE	0.09	0.001
MDD	bipolar disorder	0.979<br>[0.854, 1.121]	0.755	FALSE	FALSE	0.066	2.09E-05
Any depressive disorder	bipolar disorder	1.013<br>[0.927, 1.107]	0.777	FALSE	FALSE	0.037	7.27E-05
Suicidal ideation	bipolar disorder	0.989<br>[0.910, 1.074]	0.793	FALSE	FALSE	0.045	7.76E-05
ADHD	bipolar disorder	1.001<br>[0.946, 1.060]	0.961	FALSE	FALSE	0.042	3.38E-05
Suicidal behaviors	bipolar disorder	0.998<br>[0.919, 1.084]	0.962	FALSE	FALSE	0.044	6.12E-05

P values were adjusted for 72 tests (36 outcomes and 2 PGSs of depression and bipolar disorder). ∆McFadden's R^2 is the proportion of variance explained by PGS on KSADS diagnosis.

Supplementary Table 8. Effects of depression and bipolar PGSs on KSADS diagnosis in European-ancestry children

KSADS diagnosis	PGS	OR [95% Confidence interval]	Unadjusted *P* value	FDR-corrected *P*<.05	Bonferroni-corrected *P*<.05	McFadden's R^2	∆McFadden's R^2
Any psychiatric disorder	depression	1.13 [1.067, 1.197]	2.83E-05	TRUE	TRUE	0.025	0.002
Self-harm	depression	1.297 [1.145, 1.471]	4.40E-05	TRUE	TRUE	0.044	0.008
Any psychiatric disorder (child)	depression	1.13 [1.063, 1.202]	8.97E-05	TRUE	TRUE	0.023	0.002
Suicidal ideation	depression	1.201 [1.092, 1.321]	1.54E-04	TRUE	TRUE	0.055	0.004
Suicidal behaviors	depression	1.198 [1.090, 1.318]	1.74E-04	TRUE	TRUE	0.054	0.004
Suicidal ideation (child)	depression	1.196 [1.088, 1.314]	1.92E-04	TRUE	TRUE	0.042	0.004
Suicidal behaviors (child)	depression	1.191 [1.084, 1.308]	2.58E-04	TRUE	TRUE	0.042	0.004
Conduct/Oppositional defiant disorder	depression	1.134 [1.058, 1.216]	3.79E-04	TRUE	TRUE	0.036	0.002
Any anxiety disorder	depression	1.155 [1.066, 1.251]	4.22E-04	TRUE	TRUE	0.026	0.003
Sleep problems (child)	depression	1.128 [1.049, 1.213]	0.001	TRUE	FALSE	0.015	0.002
Generalized anxiety disorder	depression	1.2 [1.068, 1.349]	0.002	TRUE	FALSE	0.038	0.004
Conduct disorder	depression	1.282 [1.094, 1.504]	0.002	TRUE	FALSE	0.105	0.007
Sleep problems	depression	1.136 [1.046, 1.235]	0.003	TRUE	FALSE	0.023	0.002
ADHD	depression	1.107 [1.036, 1.183]	0.003	TRUE	FALSE	0.041	0.002
MDD	depression	1.254 [1.064, 1.481]	0.007	TRUE	FALSE	0.095	0.005
Separation anxiety disorder	depression	1.123 [1.032, 1.222]	0.007	TRUE	FALSE	0.028	0.002
Suicidal plan	depression	1.557 [1.122, 2.167]	0.008	TRUE	FALSE	0.201	0.015
Simple/specific phobia	depression	1.068 [1.009, 1.132]	0.023	FALSE	FALSE	0.015	0.001
PTSD	bipolar disorder	1.238 [1.022, 1.500]	0.029	FALSE	FALSE	0.13	0.005
PTSD	depression	1.24 [1.022, 1.506]	0.029	FALSE	FALSE	0.13	0.005
Suicidal attempt (child)	depression	1.331 [1.029, 1.724]	0.029	FALSE	FALSE	0.147	0.007
Any depressive disorder	depression	1.126 [1.012, 1.253]	0.03	FALSE	FALSE	0.048	0.002
MDD (child)	depression	1.219 [1.019, 1.461]	0.031	FALSE	FALSE	0.058	0.004
Psychotic disorder	depression	1.201 [1.012, 1.427]	0.036	FALSE	FALSE	0.056	0.004
Any depressive disorder (child)	depression	1.146 [1.007, 1.304]	0.039	FALSE	FALSE	0.038	0.002
Suicidal attempt (child)	bipolar disorder	1.273 [0.991, 1.634]	0.059	FALSE	FALSE	0.145	0.006
Agoraphobia	depression	1.354 [0.970, 1.890]	0.075	FALSE	FALSE	0.092	0.008
Suicidal attepmt	depression	1.412 [0.955, 2.092]	0.084	FALSE	FALSE	0.142	0.01
Eating disorder	depression	0.931 [0.856, 1.013]	0.097	FALSE	FALSE	0.012	0.001
Panic disorder	bipolar disorder	1.466 [0.921, 2.329]	0.106	FALSE	FALSE	0.197	0.012
OCD	depression	1.075 [0.983, 1.176]	0.111	FALSE	FALSE	0.032	0.001
Bipolar disorder	depression	1.089 [0.975, 1.216]	0.131	FALSE	FALSE	0.056	0.001
Agoraphobia	bipolar disorder	1.283 [0.921, 1.790]	0.141	FALSE	FALSE	0.09	0.006
Social anxiety disorder	depression	1.091 [0.971, 1.226]	0.142	FALSE	FALSE	0.025	0.001
Social anxiety disorder	bipolar disorder	0.918 [0.818, 1.030]	0.145	FALSE	FALSE	0.025	0.001
Any anxiety disorder (child)	depression	1.11 [0.958, 1.286]	0.166	FALSE	FALSE	0.048	0.001
Generalized anxiety disorder (child)	depression	1.234 [0.913, 1.671]	0.171	FALSE	FALSE	0.125	0.004
Suicidal attepmt	bipolar disorder	1.31 [0.887, 1.944]	0.175	FALSE	FALSE	0.139	0.006
Sleep problems (child)	bipolar disorder	0.955 [0.889, 1.026]	0.209	FALSE	FALSE	0.013	3.33E-04
Any anxiety disorder	bipolar disorder	0.951 [0.879, 1.029]	0.214	FALSE	FALSE	0.023	3.90E-04
Conduct/Oppositional defiant disorder	bipolar disorder	0.958 [0.895, 1.027]	0.225	FALSE	FALSE	0.034	3.12E-04
ADHD	bipolar disorder	0.961 [0.900, 1.026]	0.234	FALSE	FALSE	0.04	2.82E-04
Suicidal plan	bipolar disorder	0.837 [0.608, 1.153]	0.276	FALSE	FALSE	0.189	0.004
Suicidal plan (child)	depression	1.135 [0.904, 1.425]	0.276	FALSE	FALSE	0.099	0.002
Bipolar disorder (child)	bipolar disorder	0.855 [0.636, 1.150]	0.302	FALSE	FALSE	0.087	0.003

Any depressive disorder (child)	bipolar disorder	1.067 [0.938, 1.213]	0.325	FALSE	FALSE	0.037	0.001
Any anxiety disorder (child)	bipolar disorder	1.075 [0.930, 1.243]	0.328	FALSE	FALSE	0.048	0.001
Self-harm	bipolar disorder	1.06 [0.938, 1.199]	0.352	FALSE	FALSE	0.036	4.86E-04
Any depressive disorder	bipolar disorder	1.05 [0.945, 1.167]	0.363	FALSE	FALSE	0.047	3.83E-04
Eating disorder	bipolar disorder	1.036 [0.954, 1.127]	0.4	FALSE	FALSE	0.011	1.94E-04
MDD (child)	bipolar disorder	1.078 [0.901, 1.289]	0.412	FALSE	FALSE	0.055	0.001
Panic disorder	depression	0.837 [0.523, 1.345]	0.459	FALSE	FALSE	0.189	0.004
Simple/specific phobia	bipolar disorder	0.98 [0.926, 1.037]	0.476	FALSE	FALSE	0.015	8.37E-05
Social anxiety disorder (child)	depression	1.107 [0.835, 1.470]	0.479	FALSE	FALSE	0.103	0.001
OCD	bipolar disorder	0.97 [0.888, 1.059]	0.499	FALSE	FALSE	0.031	1.74E-04
Suicidal plan (child)	bipolar disorder	0.933 [0.745, 1.168]	0.546	FALSE	FALSE	0.098	0.001
Psychotic disorder	bipolar disorder	0.952 [0.804, 1.127]	0.566	FALSE	FALSE	0.053	4.22E-04
Any psychiatric disorder (child)	bipolar disorder	0.983 [0.925, 1.044]	0.574	FALSE	FALSE	0.021	6.77E-05
Generalized anxiety disorder	bipolar disorder	1.033 [0.921, 1.159]	0.579	FALSE	FALSE	0.034	2.00E-04
Conduct disorder	bipolar disorder	0.959 [0.818, 1.123]	0.601	FALSE	FALSE	0.099	4.93E-04
Suicidal ideation	bipolar disorder	0.976 [0.888, 1.071]	0.603	FALSE	FALSE	0.051	1.61E-04
Any psychiatric disorder	bipolar disorder	0.988 [0.933, 1.046]	0.675	FALSE	FALSE	0.023	4.36E-05
Sleep problems	bipolar disorder	0.983 [0.905, 1.067]	0.677	FALSE	FALSE	0.021	7.42E-05
Bipolar disorder (child)	depression	1.056 [0.784, 1.424]	0.721	FALSE	FALSE	0.086	0.001
Suicidal behaviors	bipolar disorder	0.985 [0.898, 1.082]	0.757	FALSE	FALSE	0.05	1.08E-04
Generalized anxiety disorder (child)	bipolar disorder	1.041 [0.774, 1.400]	0.789	FALSE	FALSE	0.121	0.001
Suicidal behaviors (child)	bipolar disorder	1.006 [0.917, 1.103]	0.901	FALSE	FALSE	0.038	6.29E-05
Separation anxiety disorder	bipolar disorder	0.997 [0.917, 1.083]	0.937	FALSE	FALSE	0.026	3.95E-05
Suicidal ideation (child)	bipolar disorder	1.002 [0.913, 1.100]	0.959	FALSE	FALSE	0.038	5.88E-05
MDD	bipolar disorder	0.998 [0.848, 1.175]	0.979	FALSE	FALSE	0.09	2.70E-04
Bipolar disorder	bipolar disorder	0.999 [0.896, 1.113]	0.981	FALSE	FALSE	0.055	9.88E-05
Social anxiety disorder (child)	bipolar disorder	0.999 [0.759, 1.318]	0.996	FALSE	FALSE	0.102	0.001

P values were adjusted for 72 tests (36 outcomes and 2 PGSs of depression and bipolar disorder). ΔMcFadden's R^2 is the proportion of variance explained by PGS on KSADS diagnosis.

Supplementary Table 9. Effects of family history of depression on KSADS diagnosis in multi-ancestry children

KSADS diagnosis	OR [95% Confidence interval]			Unadjusted *P* value			FDR-corrected *P*<.05			Bonferroni-corrected *P*<.05			McFadden's R^2	∆McFadden's R^2
	G1+/G2-	G1-/G2+	G1+/G2+	G1+/G2-	G1-/G2+	G1+/G2+	G1+/G2-	G1-/G2+	G1+/G2+	G1+/G2-	G1-/G2+	G1+/G2+		
Any psychiatric disorder	1.392 [1.190, 1.625]	2.143 [1.832, 2.505]	2.735 [2.403, 3.112]	4.17E-05	< 2.00e-16	< 2.00e-16	TRUE	TRUE	TRUE	TRUE	TRUE	TRUE	0.048	0.028
Any depressive disorder	1.684 [1.234, 2.270]	3.068 [2.354, 3.979]	3.643 [2.917, 4.553]	0.001	2.11E-15	< 2.00e-16	TRUE	TRUE	TRUE	TRUE	TRUE	TRUE	0.076	0.039
ADHD	1.324 [1.102, 1.586]	2.028 [1.696, 2.418]	2.311 [1.995, 2.676]	0.003	3.69E-14	< 2.00e-16	TRUE	TRUE	TRUE	FALSE	TRUE	TRUE	0.061	0.019
Suicidal ideation	1.385 [1.040, 1.823]	2.57 [1.999, 3.286]	2.852 [2.317, 3.509]	0.026	1.42E-12	< 2.00e-16	FALSE	TRUE	TRUE	FALSE	TRUE	TRUE	0.072	0.027
Suicidal behaviors	1.36 [1.022, 1.790]	2.526 [1.966, 3.227]	2.814 [2.288, 3.459]	0.035	3.13E-12	< 2.00e-16	FALSE	TRUE	TRUE	FALSE	TRUE	TRUE	0.071	0.027
Any anxiety disorder	1.467 [1.161, 1.841]	2.175 [1.734, 2.716]	3.134 [2.627, 3.739]	0.001	8.63E-11	< 2.00e-16	TRUE	TRUE	TRUE	FALSE	TRUE	TRUE	0.055	0.031
Conduct/Oppositional defiant disorder	1.465 [1.205, 1.774]	1.886 [1.550, 2.286]	2.538 [2.174, 2.962]	1.47E-04	5.87E-10	< 2.00e-16	TRUE	TRUE	TRUE	TRUE	TRUE	TRUE	0.058	0.021
Separation anxiety disorder	1.68 [1.318, 2.129]	2.178 [1.715, 2.750]	3.162 [2.623, 3.812]	3.95E-05	6.63E-10	< 2.00e-16	TRUE	TRUE	TRUE	TRUE	TRUE	TRUE	0.058	0.031
MDD	2.131 [1.273, 3.466]	3.736 [2.432, 5.692]	5.906 [4.184, 8.422]	0.005	8.23E-09	< 2.00e-16	TRUE	TRUE	TRUE	FALSE	TRUE	TRUE	0.123	0.058
Generalized anxiety disorder	1.437 [0.974, 2.080]	2.476 [1.747, 3.468]	4.561 [3.529, 5.916]	0.067	8.92E-07	< 2.00e-16	FALSE	TRUE	TRUE	FALSE	TRUE	TRUE	0.086	0.049
Simple/specific phobia	1.399 [1.203, 1.624]	1.491 [1.271, 1.746]	1.784 [1.566, 2.031]	1.49E-05	1.21E-06	< 2.00e-16	TRUE	TRUE	TRUE	TRUE	TRUE	TRUE	0.019	0.009
Sleep problems	1.442 [1.141, 1.811]	1.78 [1.401, 2.244]	2.479 [2.063, 2.976]	0.002	3.89E-06	< 2.00e-16	TRUE	TRUE	TRUE	FALSE	TRUE	TRUE	0.04	0.019
PTSD	1.281 [0.634, 2.382]	2.121 [1.251, 3.489]	4.669 [3.210, 6.845]	0.47	0.006	7.77E-16	FALSE	TRUE	TRUE	FALSE	FALSE	TRUE	0.142	0.044
OCD	1.353 [1.060, 1.715]	1.537 [1.207, 1.944]	1.977 [1.634, 2.389]	0.016	0.001	6.07E-12	TRUE	TRUE	TRUE	FALSE	TRUE	TRUE	0.041	0.01
Social anxiety disorder	1.838 [1.335, 2.503]	2.414 [1.757, 3.284]	2.553 [1.961, 3.319]	2.49E-04	1.64E-07	8.14E-12	TRUE	TRUE	TRUE	TRUE	TRUE	TRUE	0.045	0.021
Self-harm	1.643 [1.148, 2.315]	1.832 [1.272, 2.594]	2.535 [1.926, 3.332]	0.007	0.001	6.76E-11	TRUE	TRUE	TRUE	FALSE	FALSE	TRUE	0.049	0.017
Conduct disorder	1.703 [1.087, 2.599]	2.127 [1.422, 3.125]	2.463 [1.778, 3.404]	0.021	3.41E-04	9.57E-08	FALSE	TRUE	TRUE	FALSE	TRUE	TRUE	0.106	0.017
Bipolar disorder	1.128 [0.825, 1.518]	1.854 [1.431, 2.382]	1.833 [1.466, 2.284]	0.442	4.98E-06	1.67E-07	FALSE	TRUE	TRUE	FALSE	TRUE	TRUE	0.066	0.01
Psychotic disorder	1.882 [1.205, 2.871]	2.108 [1.362, 3.185]	2.508 [1.765, 3.548]	0.006	0.001	5.00E-07	TRUE	TRUE	TRUE	FALSE	TRUE	TRUE	0.062	0.017
Suicidal behaviors (child)	0.961 [0.734, 1.244]	1.319 [1.019, 1.690]	1.537 [1.252, 1.879]	0.767	0.036	4.68E-05	FALSE	FALSE	TRUE	FALSE	FALSE	TRUE	0.035	0.004
Suicidal attempt (child)	1.525 [0.749, 2.872]	1.684 [0.889, 3.011]	2.713 [1.691, 4.325]	0.232	0.106	4.85E-05	FALSE	FALSE	TRUE	FALSE	FALSE	TRUE	0.097	0.016
Any psychiatric disorder (child)	1.029 [0.872, 1.211]	1.227 [1.036, 1.449]	1.326 [1.154, 1.523]	0.73	0.018	7.62E-05	FALSE	TRUE	TRUE	FALSE	FALSE	TRUE	0.022	0.002
Suicidal ideation (child)	0.942 [0.719, 1.221]	1.272 [0.980, 1.636]	1.474 [1.199, 1.807]	0.658	0.07	2.70E-04	FALSE	FALSE	TRUE	FALSE	FALSE	TRUE	0.034	0.004
Panic disorder	5.172 [1.068, 25.066]	10.623 [3.061, 44.584]	7.242 [2.227, 29.352]	0.042	2.19E-04	0.001	FALSE	TRUE	TRUE	FALSE	TRUE	TRUE	0.233	0.059
Social anxiety disorder (child)	1.545 [0.659, 3.285]	2.166 [0.999, 4.377]	2.722 [1.510, 4.881]	0.299	0.05	0.001	FALSE	FALSE	TRUE	FALSE	FALSE	TRUE	0.106	0.017
Suicidal plan (child)	0.845 [0.374, 1.694]	2.091 [1.155, 3.628]	2.156 [1.324, 3.477]	0.652	0.016	0.002	FALSE	TRUE	TRUE	FALSE	FALSE	FALSE	0.084	0.014
Any anxiety disorder (child)	1.43 [0.962, 2.078]	1.534 [1.032, 2.229]	1.645 [1.195, 2.248]	0.076	0.035	0.003	FALSE	FALSE	TRUE	FALSE	FALSE	FALSE	0.049	0.005
Any depressive disorder (child)	0.947 [0.659, 1.330]	1.21 [0.863, 1.665]	1.407 [1.073, 1.831]	0.76	0.263	0.014	FALSE	FALSE	TRUE	FALSE	FALSE	FALSE	0.036	0.003
Generalized anxiety disorder (child)	1.393 [0.570, 3.017]	1.538 [0.660, 3.226]	2.063 [1.132, 3.699]	0.443	0.3	0.019	FALSE	FALSE	TRUE	FALSE	FALSE	FALSE	0.106	0.008
Agoraphobia	1.472 [0.504, 3.667]	2.83 [1.223, 6.154]	2.387 [1.119, 4.982]	0.451	0.017	0.025	FALSE	TRUE	TRUE	FALSE	FALSE	FALSE	0.091	0.016
Sleep problems (child)	1.091 [0.900, 1.317]	1.079 [0.879, 1.318]	1.179 [0.999, 1.389]	0.371	0.462	0.051	FALSE	FALSE	FALSE	FALSE	FALSE	FALSE	0.011	0.001
Suicidal attepmt	1.366 [0.413, 3.700]	2.522 [0.980, 5.952]	1.962 [0.845, 4.351]	0.578	0.055	0.113	FALSE	FALSE	FALSE	FALSE	FALSE	FALSE	0.112	0.011

Eating disorder	0.888 [0.699, 1.118]	1.251 [0.991, 1.567]	1.126 [0.923, 1.368]	0.316	0.06	0.239	FALSE	FALSE	FALSE	FALSE	FALSE	FALSE	0.008	0.001
Suicidal plan	1.686 [0.721, 3.571]	2.649 [1.292, 5.146]	1.478 [0.720, 2.887]	0.215	0.009	0.276	FALSE	TRUE	FALSE	FALSE	FALSE	FALSE	0.137	0.011
Bipolar disorder (child)	1.881 [0.933, 3.560]	1.181 [0.521, 2.384]	1.393 [0.726, 2.539]	0.076	0.669	0.307	FALSE	FALSE	FALSE	FALSE	FALSE	FALSE	0.076	0.005
MDD (child)	0.882 [0.536, 1.385]	1.092 [0.693, 1.662]	1.137 [0.782, 1.626]	0.598	0.694	0.494	FALSE	FALSE	FALSE	FALSE	FALSE	FALSE	0.05	0.001

Regression of four risk levels of family history from the two generations: no depression history (G1−/G2−; reference level), only grandparent (G1+/G2−), only parent (G1−/G2+), and both generations (G1+/G2+). ∆McFadden's R^2 is the proportion of variance explained by family history of depression on KSADS diagnosis. *P* values were adjusted for 36 tests.

Supplementary Table 10. Effects of family history of depression on KSADS diagnosis in European-ancestry children

KSADS diagnosis	OR [95% Confidence interval]			Unadjusted *P* value			FDR-corrected *P*<.05			Bonferroni-corrected *P*<.05			McFadden's R^2	ΔMcFadden's R^2
	G1+/G2-	G1-/G2+	G1+/G2+	G1+/G2-	G1-/G2+	G1+/G2+	G1+/G2-	G1-/G2+	G1+/G2+	G1+/G2-	G1-/G2+	G1+/G2+		
Any psychiatric disorder	1.331 [1.122, 1.575]	1.944 [1.614, 2.337]	2.529 [2.188, 2.923]	0.001	5.34E-12	< 2.00e-16	TRUE	TRUE	TRUE	TRUE	TRUE	TRUE	0.047	0.024
Any depressive disorder	1.77 [1.245, 2.489]	3.16 [2.270, 4.370]	3.875 [2.974, 5.066]	0.002	5.47E-11	< 2.00e-16	TRUE	TRUE	TRUE	FALSE	TRUE	TRUE	0.088	0.041
ADHD	1.201 [0.982, 1.464]	1.948 [1.581, 2.392]	2.179 [1.848, 2.567]	0.074	8.50E-10	< 2.00e-16	FALSE	TRUE	TRUE	FALSE	TRUE	TRUE	0.057	0.017
MDD	2.368 [1.304, 4.208]	4.435 [2.597, 7.556]	6.331 [4.121, 9.971]	0.005	1.32E-07	< 2.00e-16	TRUE	TRUE	TRUE	FALSE	TRUE	TRUE	0.148	0.059
Any anxiety disorder	1.385 [1.079, 1.765]	1.941 [1.495, 2.503]	2.909 [2.397, 3.531]	0.011	1.23E-06	< 2.00e-16	TRUE	TRUE	TRUE	FALSE	TRUE	TRUE	0.051	0.028
Separation anxiety disorder	1.513 [1.167, 1.950]	1.978 [1.506, 2.577]	2.738 [2.229, 3.363]	0.002	1.72E-06	< 2.00e-16	TRUE	TRUE	TRUE	FALSE	TRUE	TRUE	0.05	0.024
Conduct/Oppositional defiant disorder	1.328 [1.077, 1.630]	1.613 [1.282, 2.017]	2.277 [1.919, 2.701]	0.008	5.61E-05	< 2.00e-16	TRUE	TRUE	TRUE	FALSE	TRUE	TRUE	0.051	0.017
Generalized anxiety disorder	1.286 [0.850, 1.903]	1.972 [1.308, 2.913]	4.284 [3.251, 5.673]	0.228	0.001	< 2.00e-16	FALSE	TRUE	TRUE	FALSE	FALSE	TRUE	0.082	0.048
Sleep problems	1.464 [1.143, 1.865]	1.593 [1.203, 2.089]	2.36 [1.926, 2.890]	0.003	0.001	3.33E-16	TRUE	TRUE	TRUE	FALSE	FALSE	TRUE	0.038	0.017
Suicidal ideation	1.176 [0.857, 1.591]	2.295 [1.722, 3.038]	2.468 [1.961, 3.105]	0.31	< 2.00e-16	2.79E-14	FALSE	TRUE	TRUE	FALSE	TRUE	TRUE	0.072	0.022
Suicidal behaviors	1.16 [0.846, 1.569]	2.267 [1.701, 2.998]	2.451 [1.949, 3.080]	0.35	< 2.00e-16	3.51E-14	FALSE	TRUE	TRUE	FALSE	TRUE	TRUE	0.072	0.022
Simple/specific phobia	1.394 [1.183, 1.640]	1.365 [1.127, 1.647]	1.729 [1.495, 2.000]	< 2.00e-16	0.002	2.86E-13	TRUE	TRUE	TRUE	TRUE	FALSE	TRUE	0.023	0.008
PTSD	1.108 [0.482, 2.284]	2.284 [1.210, 4.153]	3.983 [2.539, 6.337]	0.796	0.012	1.49E-09	FALSE	TRUE	TRUE	FALSE	FALSE	TRUE	0.162	0.037
OCD	1.354 [1.037, 1.754]	1.514 [1.127, 2.011]	1.876 [1.506, 2.333]	0.026	0.006	2.91E-08	FALSE	TRUE	TRUE	FALSE	FALSE	TRUE	0.04	0.009
Social anxiety disorder	1.719 [1.219, 2.398]	2.314 [1.610, 3.282]	2.281 [1.702, 3.055]	0.002	< 2.00e-16	5.13E-08	TRUE	TRUE	TRUE	FALSE	TRUE	TRUE	0.04	0.017
Self-harm	1.529 [1.042, 2.205]	1.738 [1.153, 2.565]	2.271 [1.680, 3.067]	0.03	0.009	1.34E-07	FALSE	TRUE	TRUE	FALSE	FALSE	TRUE	0.05	0.014
Conduct disorder	1.436 [0.862, 2.318]	1.232 [0.681, 2.111]	2.213 [1.516, 3.232]	0.16	0.475	4.49E-05	FALSE	FALSE	TRUE	FALSE	FALSE	TRUE	0.11	0.012
Psychotic disorder	1.697 [1.011, 2.771]	2.22 [1.297, 3.679]	2.316 [1.522, 3.514]	0.045	0.004	1.07E-04	FALSE	TRUE	TRUE	FALSE	FALSE	TRUE	0.067	0.015
Social anxiety disorder (child)	2.036 [0.832, 4.659]	2.41 [0.939, 5.678]	3.592 [1.842, 7.143]	0.115	0.066	1.94E-04	FALSE	FALSE	TRUE	FALSE	FALSE	TRUE	0.128	0.026
Bipolar disorder	1.014 [0.706, 1.428]	1.755 [1.258, 2.416]	1.593 [1.216, 2.077]	0.936	0.001	0.001	FALSE	TRUE	TRUE	FALSE	TRUE	TRUE	0.062	0.007
Suicidal behaviors (child)	0.982 [0.733, 1.300]	1.359 [1.005, 1.817]	1.465 [1.160, 1.844]	0.901	0.047	0.001	FALSE	FALSE	TRUE	FALSE	FALSE	FALSE	0.042	0.004
Any anxiety disorder (child)	1.575 [1.021, 2.378]	1.59 [0.988, 2.485]	1.789 [1.245, 2.554]	0.04	0.056	0.002	FALSE	FALSE	TRUE	FALSE	FALSE	FALSE	0.054	0.008
Suicidal ideation (child)	0.982 [0.733, 1.300]	1.341 [0.989, 1.795]	1.446 [1.144, 1.822]	0.901	0.058	0.002	FALSE	FALSE	TRUE	FALSE	FALSE	FALSE	0.042	0.004
Suicidal attempt (child)	1.42 [0.607, 3.018]	1.439 [0.608, 3.087]	2.409 [1.344, 4.318]	0.398	0.387	0.003	FALSE	FALSE	TRUE	FALSE	FALSE	FALSE	0.153	0.013
Any psychiatric disorder (child)	1.064 [0.889, 1.270]	1.245 [1.018, 1.517]	1.259 [1.075, 1.472]	0.494	0.033	0.004	FALSE	FALSE	TRUE	FALSE	FALSE	FALSE	0.022	0.002
Panic disorder	4.782 [0.930, 28.913]	10.375 [2.578, 58.154]	6.444 [1.682, 34.950]	0.06	0.001	0.006	FALSE	TRUE	TRUE	FALSE	TRUE	FALSE	0.239	0.054
Suicidal plan (child)	0.818 [0.339, 1.735]	1.954 [0.980, 3.694]	1.978 [1.153, 3.372]	0.619	0.057	0.014	FALSE	FALSE	TRUE	FALSE	FALSE	FALSE	0.109	0.012
Any depressive disorder (child)	0.799 [0.517, 1.197]	0.973 [0.615, 1.484]	1.462 [1.070, 1.986]	0.284	0.901	0.018	FALSE	FALSE	TRUE	FALSE	FALSE	FALSE	0.041	0.005
Generalized anxiety disorder (child)	1.35 [0.506, 3.159]	1.324 [0.448, 3.300]	1.909 [0.962, 3.745]	0.524	0.584	0.064	FALSE	FALSE	FALSE	FALSE	FALSE	FALSE	0.128	0.007
MDD (child)	0.724 [0.382, 1.274]	0.938 [0.490, 1.671]	1.327 [0.858, 2.023]	0.273	0.835	0.2	FALSE	FALSE	FALSE	FALSE	FALSE	FALSE	0.058	0.004
Agoraphobia	1.487 [0.500, 3.844]	3.359 [1.396, 7.755]	1.746 [0.723, 4.064]	0.45	0.008	0.208	FALSE	TRUE	FALSE	FALSE	FALSE	FALSE	0.102	0.017
Sleep problems (child)	1.126 [0.914, 1.381]	1.115 [0.875, 1.411]	1.115 [0.923, 1.343]	0.262	0.374	0.256	FALSE	FALSE	FALSE	FALSE	FALSE	FALSE	0.013	4.93E-04

Suicidal plan	1.291 [0.442, 3.222]	2.219 [0.855, 5.238]	1.574 [0.696, 3.421]	0.615	0.097	0.268	FALSE	FALSE	FALSE	FALSE	FALSE	FALSE	0.193	0.008
Bipolar disorder (child)	2.353 [1.082, 4.907]	1.201 [0.372, 3.115]	1.52 [0.701, 3.155]	0.032	0.734	0.279	FALSE	FALSE	FALSE	FALSE	FALSE	FALSE	0.095	0.01
Suicidal attepmt	1.119 [0.283, 3.408]	2.185 [0.707, 5.968]	1.52 [0.571, 3.806]	0.856	0.163	0.387	FALSE	FALSE	FALSE	FALSE	FALSE	FALSE	0.14	0.007
Eating disorder	0.877 [0.680, 1.119]	1.004 [0.756, 1.316]	0.982 [0.785, 1.221]	0.294	0.977	0.871	FALSE	FALSE	FALSE	FALSE	FALSE	FALSE	0.011	3.29E-04

Regression of four risk levels of family history from the two generations: no depression history (G1−/G2−; reference level), only grandparent (G1+/G2−), only parent (G1−/G2+), and both generations (G1+/G2+). ΔMcFadden's R^2 is the proportion of variance explained by family history of depression on KSADS diagnosis. *P* values were adjusted for 36 tests.

Supplementary Table 11. Effects of family history of depression and depression PGS on KSADS diagnosis in multi-ancestry children

KSADS diagnosis	OR [95% Confidence interval]				Unadjusted *P* value				FDR-corrected *P*<.05				McFadden's R^2	∆McFadden's R^2	∆McFadden's R^2 (FH)
	PGS	G1+/G2-	G1-/G2+	G1+/G2+	PGS	G1+/G2-	G1-/G2+	G1+/G2+	PGS	G1+/G2-	G1-/G2+	G1+/G2+			
Any psychiatric disorder	1.112 [1.057, 1.170]	1.381 [1.180, 1.613]	2.134 [1.823, 2.494]	2.702 [2.374, 3.076]	3.93E-05	6.34E-05	< 2.00E-16	< 2.00E-16	TRUE	TRUE	TRUE	TRUE	0.05	0.03	0.028
Any psychiatric disorder (child)	1.111 [1.054, 1.171]	1.021 [0.865, 1.201]	1.221 [1.030, 1.442]	1.308 [1.138, 1.502]	8.50E-05	0.806	0.021	1.73E-04	TRUE	FALSE	TRUE	TRUE	0.023	0.004	0.002
Conduct/Oppositional defiant disorder	1.131 [1.062, 1.204]	1.455 [1.197, 1.762]	1.879 [1.544, 2.277]	2.504 [2.144, 2.923]	1.16E-04	1.93E-04	7.66E-10	< 2.00E-16	TRUE	TRUE	TRUE	TRUE	0.06	0.024	0.021
Conduct disorder	1.258 [1.100, 1.439]	1.687 [1.077, 2.575]	2.107 [1.407, 3.098]	2.39 [1.723, 3.306]	0.001	0.023	4.09E-04	2.65E-07	TRUE	FALSE	TRUE	TRUE	0.112	0.022	0.017
Any anxiety disorder	1.132 [1.052, 1.217]	1.453 [1.150, 1.825]	2.163 [1.723, 2.701]	3.092 [2.591, 3.690]	0.001	0.002	1.20E-10	< 2.00E-16	TRUE	TRUE	TRUE	TRUE	0.057	0.033	0.031
Suicidal ideation (child)	1.139 [1.051, 1.235]	0.933 [0.712, 1.209]	1.265 [0.973, 1.626]	1.45 [1.178, 1.777]	0.002	0.608	0.078	0.001	TRUE	FALSE	FALSE	TRUE	0.036	0.006	0.004
Sleep problems (child)	1.102 [1.036, 1.173]	1.083 [0.893, 1.307]	1.074 [0.875, 1.311]	1.164 [0.986, 1.371]	0.002	0.416	0.491	0.073	TRUE	FALSE	FALSE	FALSE	0.013	0.002	0.001
ADHD	1.095 [1.033, 1.161]	1.317 [1.096, 1.578]	2.021 [1.690, 2.411]	2.286 [1.972, 2.647]	0.002	0.003	4.87E-14	< 2.00E-16	TRUE	TRUE	TRUE	TRUE	0.062	0.02	0.019
Self-harm	1.184 [1.058, 1.324]	1.625 [1.135, 2.290]	1.818 [1.262, 2.574]	2.482 [1.885, 3.264]	0.003	0.009	0.002	1.89E-10	TRUE	TRUE	TRUE	TRUE	0.052	0.021	0.017
Separation anxiety disorder	1.121 [1.038, 1.210]	1.668 [1.308, 2.114]	2.168 [1.707, 2.738]	3.118 [2.585, 3.760]	0.004	5.03E-05	8.37E-10	< 2.00E-16	TRUE	TRUE	TRUE	TRUE	0.06	0.032	0.031
Suicidal behaviors (child)	1.127 [1.040, 1.221]	0.953 [0.728, 1.233]	1.311 [1.013, 1.680]	1.513 [1.232, 1.851]	0.004	0.718	0.04	8.95E-05	TRUE	FALSE	FALSE	TRUE	0.037	0.006	0.004
Generalized anxiety disorder	1.158 [1.040, 1.289]	1.422 [0.963, 2.058]	2.461 [1.736, 3.448]	4.49 [3.473, 5.826]	0.008	0.076	1.06E-06	< 2.00E-16	TRUE	FALSE	TRUE	TRUE	0.089	0.051	0.049
Sleep problems	1.106 [1.027, 1.192]	1.432 [1.133, 1.799]	1.77 [1.394, 2.233]	2.451 [2.039, 2.943]	0.008	0.003	4.75E-06	< 2.00E-16	TRUE	TRUE	TRUE	TRUE	0.042	0.02	0.019
Suicidal ideation	1.119 [1.029, 1.218]	1.376 [1.033, 1.812]	2.557 [1.988, 3.269]	2.813 [2.284, 3.463]	0.009	0.029	1.90E-12	< 2.00E-16	TRUE	FALSE	TRUE	TRUE	0.074	0.029	0.027
Suicidal behaviors	1.115 [1.025, 1.213]	1.351 [1.016, 1.779]	2.512 [1.955, 3.211]	2.777 [2.257, 3.415]	0.011	0.039	4.16E-12	< 2.00E-16	TRUE	FALSE	TRUE	TRUE	0.073	0.028	0.027
Any depressive disorder (child)	1.128 [1.015, 1.253]	0.938 [0.652, 1.318]	1.203 [0.858, 1.655]	1.383 [1.055, 1.801]	0.025	0.718	0.278	0.019	FALSE	FALSE	FALSE	TRUE	0.038	0.004	0.003
Psychotic disorder	1.171 [1.015, 1.351]	1.857 [1.188, 2.833]	2.086 [1.348, 3.153]	2.457 [1.729, 3.478]	0.031	0.007	0.001	8.95E-07	FALSE	TRUE	TRUE	TRUE	0.065	0.019	0.017
Bipolar disorder	1.09 [0.997, 1.192]	1.119 [0.818, 1.507]	1.844 [1.424, 2.370]	1.811 [1.448, 2.257]	0.058	0.472	5.99E-06	2.99E-07	FALSE	FALSE	TRUE	TRUE	0.067	0.011	0.01
Simple/specific phobia	1.045 [0.995, 1.098]	1.393 [1.198, 1.618]	1.487 [1.267, 1.741]	1.774 [1.557, 2.020]	0.081	1.86E-05	1.43E-06	< 2.00E-16	FALSE	TRUE	TRUE	TRUE	0.019	0.01	0.009
MDD (child)	1.125 [0.977, 1.296]	0.873 [0.531, 1.371]	1.085 [0.688, 1.650]	1.118 [0.769, 1.600]	0.1	0.567	0.717	0.552	FALSE	FALSE	FALSE	FALSE	0.052	0.002	0.001
PTSD	1.145 [0.973, 1.348]	1.269 [0.629, 2.360]	2.104 [1.240, 3.461]	4.582 [3.147, 6.720]	0.104	0.486	0.007	2.00E-15	FALSE	FALSE	TRUE	TRUE	0.144	0.046	0.044
Social anxiety disorder	1.081 [0.973, 1.200]	1.828 [1.328, 2.490]	2.404 [1.749, 3.270]	2.53 [1.943, 3.289]	0.145	2.81E-04	1.89E-07	1.35E-11	FALSE	TRUE	TRUE	TRUE	0.045	0.021	0.021
Eating disorder	0.948 [0.881, 1.020]	0.892 [0.702, 1.124]	1.255 [0.994, 1.573]	1.135 [0.930, 1.379]	0.15	0.336	0.056	0.212	FALSE	FALSE	FALSE	FALSE	0.008	0.002	0.001
Any depressive disorder	1.066 [0.973, 1.168]	1.674 [1.227, 2.257]	3.055 [2.344, 3.962]	3.609 [2.888, 4.512]	0.169	0.001	2.78E-15	< 2.00E-16	FALSE	TRUE	TRUE	TRUE	0.077	0.04	0.039
Panic disorder	0.76 [0.498, 1.153]	5.345 [1.101, 25.941]	11.049 [3.189, 46.268]	7.466 [2.289, 30.319]	0.198	0.038	1.70E-04	0.001	FALSE	FALSE	TRUE	TRUE	0.241	0.067	0.059
Any anxiety disorder (child)	1.074 [0.947, 1.218]	1.42 [0.955, 2.064]	1.525 [1.025, 2.216]	1.63 [1.183, 2.228]	0.266	0.082	0.038	0.003	FALSE	FALSE	FALSE	TRUE	0.05	0.006	0.005
MDD	1.069 [0.929, 1.229]	2.117 [1.265, 3.443]	3.716 [2.419, 5.661]	5.845 [4.139, 8.339]	0.352	0.005	9.43E-09	< 2.00E-16	FALSE	TRUE	TRUE	TRUE	0.124	0.058	0.058

Suicidal plan	1.094<br>[0.845, 1.415]	1.665<br>[0.712, 3.527]	2.613<br>[1.273, 5.080]	1.454<br>[0.708, 2.842]	0.497	0.225	0.01	0.297	FALSE	FALSE	TRUE	FALSE	0.138	0.012	0.011
Suicidal attempt (child)	1.067<br>[0.870, 1.308]	1.513<br>[0.743, 2.850]	1.675<br>[0.884, 2.996]	2.682<br>[1.670, 4.281]	0.534	0.241	0.11	6.09E-05	FALSE	FALSE	FALSE	TRUE	0.098	0.017	0.016
Agoraphobia	1.098<br>[0.816, 1.475]	1.457<br>[0.499, 3.631]	2.803<br>[1.212, 6.096]	2.348<br>[1.099, 4.910]	0.537	0.463	0.017	0.028	FALSE	FALSE	TRUE	TRUE	0.093	0.017	0.016
Suicidal plan (child)	1.053<br>[0.864, 1.284]	0.842<br>[0.373, 1.688]	2.081<br>[1.149, 3.612]	2.139<br>[1.313, 3.452]	0.609	0.645	0.017	0.003	FALSE	FALSE	TRUE	TRUE	0.085	0.014	0.014
Social anxiety disorder (child)	1.063<br>[0.828, 1.363]	1.539<br>[0.657, 3.271]	2.152<br>[0.993, 4.352]	2.702<br>[1.498, 4.846]	0.631	0.303	0.052	0.001	FALSE	FALSE	FALSE	TRUE	0.107	0.018	0.017
OCD	1.018<br>[0.943, 1.100]	1.352<br>[1.058, 1.712]	1.536<br>[1.205, 1.942]	1.972<br>[1.629, 2.383]	0.642	0.016	0.001	7.76E-12	FALSE	TRUE	TRUE	TRUE	0.041	0.01	0.01
Generalized anxiety disorder (child)	1.059<br>[0.820, 1.365]	1.384<br>[0.565, 2.997]	1.526<br>[0.654, 3.203]	2.05<br>[1.124, 3.676]	0.661	0.452	0.309	0.02	FALSE	FALSE	FALSE	TRUE	0.107	0.009	0.008
Suicidal attepmt	1.06<br>[0.759, 1.478]	1.356<br>[0.410, 3.673]	2.498<br>[0.970, 5.903]	1.943<br>[0.837, 4.314]	0.734	0.587	0.057	0.118	FALSE	FALSE	FALSE	FALSE	0.113	0.012	0.011
Bipolar disorder (child)	1.03<br>[0.812, 1.305]	1.873<br>[0.929, 3.546]	1.176<br>[0.519, 2.375]	1.385<br>[0.721, 2.528]	0.81	0.078	0.677	0.316	FALSE	FALSE	FALSE	FALSE	0.076	0.005	0.005

ΔMcFadden's R^2 is the proportion of variance explained by family history of depression and PGS on KSADS diagnosis. ΔMcFadden's R^2 (FH) is the proportion of variance explained by family history of depression on KSADS diagnosis in multi-ancestry children from Supplementary Table 7. *P* values were adjusted for 36 tests.

Supplementary Table 12. Effects of family history of depression and depression PGS on KSADS diagnosis in European-ancestry children

KSADS Diagnosis	OR [95% Confidence interval]				Unadjusted *P* value				FDR-corrected *P*<.05				McFadden's R^2	∆McFadden's R^2	∆McFadden's R^2 (FH)
	PGS	G1+/G2-	G1-/G2+	G1+/G2+	PGS	G1+/G2-	G1-/G2+	G1+/G2+	PGS	G1+/G2-	G1-/G2+	G1+/G2+			
Self-harm	1.274 [1.124, 1.444]	1.509 [1.029, 2.178]	1.725 [1.143, 2.546]	2.2 [1.626, 2.974]	1.42E-04	0.036	0.01	4.28E-07	TRUE	FALSE	TRUE	TRUE	0.057	0.021	0.014
Any psychiatric disorder (child)	1.125 [1.058, 1.196]	1.056 [0.882, 1.260]	1.241 [1.014, 1.512]	1.238 [1.057, 1.449]	1.59E-04	0.554	0.036	0.008	TRUE	FALSE	FALSE	TRUE	0.025	0.004	0.002
Suicidal ideation (child)	1.187 [1.081, 1.305]	0.972 [0.725, 1.286]	1.337 [0.986, 1.789]	1.414 [1.118, 1.783]	3.30E-04	0.843	0.062	0.004	TRUE	FALSE	FALSE	TRUE	0.046	0.008	0.004
Any psychiatric disorder	1.111 [1.049, 1.178]	1.322 [1.114, 1.564]	1.938 [1.609, 2.330]	2.497 [2.160, 2.886]	3.52E-04	0.001	6.85E-12	< 2.00E-16	TRUE	TRUE	TRUE	TRUE	0.049	0.026	0.024
Suicidal behaviors (child)	1.182 [1.076, 1.298]	0.972 [0.725, 1.287]	1.355 [1.001, 1.810]	1.433 [1.134, 1.805]	4.51E-04	0.845	0.049	0.003	TRUE	FALSE	FALSE	TRUE	0.046	0.008	0.004
Suicidal ideation	1.179 [1.072, 1.297]	1.167 [0.851, 1.579]	2.286 [1.715, 3.026]	2.415 [1.918, 3.041]	0.001	0.333	4.70E-08	1.27E-13	TRUE	FALSE	TRUE	TRUE	0.076	0.025	0.022
Suicidal behaviors	1.177 [1.070, 1.294]	1.151 [0.840, 1.558]	2.258 [1.694, 2.987]	2.399 [1.907, 3.018]	0.001	0.375	7.01E-08	1.58E-13	TRUE	FALSE	TRUE	TRUE	0.075	0.025	0.022
Sleep problems (child)	1.125 [1.047, 1.210]	1.117 [0.906, 1.370]	1.111 [0.871, 1.405]	1.097 [0.908, 1.322]	0.001	0.298	0.392	0.337	TRUE	FALSE	FALSE	FALSE	0.015	0.003	4.93E-04
Conduct/Oppositional defiant disorder	1.117 [1.042, 1.198]	1.321 [1.071, 1.621]	1.609 [1.279, 2.013]	2.247 [1.894, 2.666]	0.002	0.01	6.10E-05	< 2.00E-16	TRUE	TRUE	TRUE	TRUE	0.053	0.019	0.017
Any anxiety disorder	1.13 [1.043, 1.225]	1.373 [1.070, 1.751]	1.934 [1.489, 2.495]	2.865 [2.360, 3.479]	0.003	0.013	1.40E-06	< 2.00E-16	TRUE	FALSE	TRUE	TRUE	0.053	0.03	0.028
Conduct disorder	1.257 [1.072, 1.474]	1.433 [0.860, 2.313]	1.23 [0.680, 2.108]	2.145 [1.466, 3.137]	0.005	0.162	0.479	9.42E-05	TRUE	FALSE	FALSE	TRUE	0.116	0.018	0.012
Sleep problems	1.116 [1.027, 1.214]	1.454 [1.135, 1.852]	1.585 [1.197, 2.080]	2.328 [1.899, 2.852]	0.01	0.003	0.002	9.99E-16	TRUE	TRUE	TRUE	TRUE	0.04	0.019	0.017
ADHD	1.091 [1.021, 1.166]	1.196 [0.977, 1.457]	1.944 [1.578, 2.388]	2.154 [1.827, 2.538]	0.011	0.082	9.64E-10	< 2.00E-16	TRUE	FALSE	TRUE	TRUE	0.058	0.019	0.017
Suicidal plan	1.533 [1.104, 2.136]	1.208 [0.414, 3.013]	2.133 [0.815, 5.060]	1.441 [0.635, 3.139]	0.011	0.708	0.117	0.372	TRUE	FALSE	FALSE	FALSE	0.207	0.022	0.008
Generalized anxiety disorder	1.164 [1.035, 1.309]	1.274 [0.842, 1.885]	1.966 [1.305, 2.905]	4.208 [3.192, 5.575]	0.011	0.246	0.002	< 2.00E-16	TRUE	FALSE	TRUE	TRUE	0.085	0.05	0.048
Separation anxiety disorder	1.098 [1.009, 1.195]	1.505 [1.160, 1.940]	1.973 [1.503, 2.571]	2.702 [2.199, 3.320]	0.031	0.002	1.85E-06	< 2.00E-16	FALSE	TRUE	TRUE	TRUE	0.052	0.025	0.024
MDD	1.197 [1.014, 1.415]	2.332 [1.284, 4.145]	4.415 [2.586, 7.521]	6.146 [3.996, 9.687]	0.033	0.006	1.41E-07	< 2.00E-16	FALSE	TRUE	TRUE	TRUE	0.152	0.063	0.059
MDD (child)	1.212 [1.013, 1.451]	0.716 [0.378, 1.260]	0.932 [0.487, 1.661]	1.297 [0.838, 1.979]	0.035	0.257	0.82	0.24	FALSE	FALSE	FALSE	FALSE	0.062	0.008	0.004
Suicidal attempt (child)	1.296 [1.000, 1.680]	1.387 [0.592, 2.946]	1.418 [0.599, 3.041]	2.302 [1.282, 4.133]	0.05	0.431	0.406	0.006	FALSE	FALSE	FALSE	TRUE	0.159	0.019	0.013
Any depressive disorder (child)	1.137 [1.000, 1.293]	0.794 [0.513, 1.188]	0.97 [0.614, 1.480]	1.439 [1.052, 1.955]	0.051	0.268	0.892	0.023	FALSE	FALSE	FALSE	TRUE	0.043	0.007	0.005
Psychotic disorder	1.178 [0.993, 1.399]	1.675 [0.998, 2.735]	2.209 [1.290, 3.659]	2.259 [1.483, 3.431]	0.06	0.051	0.005	1.74E-04	FALSE	FALSE	TRUE	TRUE	0.07	0.018	0.015
Simple/specific phobia	1.055 [0.997, 1.118]	1.388 [1.178, 1.634]	1.361 [1.125, 1.643]	1.716 [1.483, 1.986]	0.065	1.01E-04	0.002	6.63E-13	FALSE	TRUE	TRUE	TRUE	0.023	0.009	0.008
PTSD	1.2 [0.988, 1.459]	1.104 [0.480, 2.276]	2.28 [1.209, 4.143]	3.889 [2.476, 6.194]	0.067	0.803	0.012	3.06E-09	FALSE	FALSE	TRUE	TRUE	0.166	0.041	0.037
Agoraphobia	1.334 [0.955, 1.864]	1.443 [0.485, 3.727]	3.284 [1.364, 7.581]	1.664 [0.687, 3.885]	0.091	0.484	0.009	0.251	FALSE	FALSE	TRUE	FALSE	0.109	0.025	0.017
Suicidal attepmt	1.387 [0.939, 2.055]	1.084 [0.275, 3.293]	2.08 [0.668, 5.706]	1.444 [0.542, 3.622]	0.1	0.897	0.193	0.447	FALSE	FALSE	FALSE	FALSE	0.149	0.017	0.007
Eating disorder	0.933 [0.858, 1.014]	0.882 [0.684, 1.126]	1.007 [0.759, 1.321]	0.992 [0.793, 1.234]	0.102	0.317	0.958	0.942	FALSE	FALSE	FALSE	FALSE	0.012	0.001	3.29E-04
Any depressive disorder	1.092 [0.981, 1.217]	1.758 [1.236, 2.473]	3.152 [2.265, 4.360]	3.825 [2.934, 5.003]	0.107	0.002	5.94E-11	< 2.00E-16	FALSE	TRUE	TRUE	TRUE	0.089	0.042	0.041

Bipolar disorder	1.078 [0.965, 1.204]	1.009 [0.702, 1.420]	1.75 [1.254, 2.408]	1.575 [1.202, 2.055]	0.182	0.961	0.001	0.001	FALSE	FALSE	TRUE	TRUE	0.063	0.008	0.007
OCD	1.06 [0.970, 1.160]	1.349 [1.033, 1.748]	1.511 [1.124, 2.007]	1.861 [1.493, 2.315]	0.2	0.028	0.007	4.65E-08	FALSE	FALSE	TRUE	TRUE	0.04	0.01	0.009
Generalized anxiety disorder (child)	1.217 [0.901, 1.647]	1.322 [0.496, 3.096]	1.3 [0.438, 3.247]	1.866 [0.939, 3.663]	0.201	0.553	0.609	0.074	FALSE	FALSE	FALSE	FALSE	0.132	0.011	0.007
Any anxiety disorder (child)	1.094 [0.945, 1.268]	1.564 [1.014, 2.362]	1.581 [0.982, 2.470]	1.766 [1.229, 2.524]	0.229	0.043	0.059	0.002	FALSE	FALSE	FALSE	TRUE	0.055	0.009	0.008
Social anxiety disorder	1.072 [0.954, 1.205]	1.71 [1.213, 2.386]	2.308 [1.606, 3.273]	2.259 [1.684, 3.026]	0.241	0.003	1.18E-05	7.67E-08	FALSE	TRUE	TRUE	TRUE	0.041	0.018	0.017
Suicidal plan (child)	1.117 [0.890, 1.403]	0.815 [0.338, 1.726]	1.947 [0.976, 3.679]	1.949 [1.135, 3.324]	0.339	0.61	0.058	0.016	FALSE	FALSE	FALSE	TRUE	0.111	0.014	0.012
Panic disorder	0.814 [0.511, 1.290]	4.87 [0.944, 29.515]	10.486 [2.602, 58.893]	6.6 [1.711, 35.844]	0.383	0.058	0.001	0.005	FALSE	FALSE	TRUE	TRUE	0.244	0.059	0.054
Social anxiety disorder (child)	1.071 [0.807, 1.424]	2.024 [0.827, 4.631]	2.394 [0.932, 5.638]	3.55 [1.819, 7.067]	0.636	0.118	0.068	2.26E-04	FALSE	FALSE	FALSE	TRUE	0.129	0.027	0.026
Bipolar disorder (child)	1.038 [0.771, 1.400]	2.341 [1.076, 4.884]	1.196 [0.371, 3.103]	1.507 [0.693, 3.136]	0.808	0.033	0.74	0.291	FALSE	FALSE	FALSE	FALSE	0.096	0.011	0.01

ΔMcFadden's R^2 is the proportion of variance explained by family history of depression and PGS on KSADS diagnosis. ΔMcFadden's R^2 (FH) are the proportion of variances explained family history of depression on KSADS diagnosis in European-ancestry children from Supplementary Table 8. P values were adjusted for 36 tests.
